# Loss of *Trp53* results in a hypoactive T cell phenotype accompanied by reduced pro-inflammatory signaling in a syngeneic orthotopic mouse model of ovarian high-grade serous carcinoma

**DOI:** 10.18632/oncotarget.28768

**Published:** 2025-09-22

**Authors:** Jacob Haagsma, Yudith Ramos Valdes, Xuejin Ou, Rasheduzzaman Rashu, S.M. Mansour Haeryfar, Jim Petrik, Trevor G. Shepherd

**Affiliations:** ^1^The Mary and John Knight Translational Ovarian Cancer Research Unit, Verspeeten Family Cancer Centre, London, ON, Canada; ^2^Department of Anatomy and Cell Biology, Schulich School of Medicine and Dentistry, Western University, London, ON, Canada; ^3^Department of Microbiology and Immunology, Schulich School of Medicine and Dentistry, Western University, London, ON, Canada; ^4^Division of Thoracic Tumor Multimodality Treatment, Cancer Center, West China Hospital, Chengdu, China; ^5^Department of Oncology, Schulich School of Medicine and Dentistry, Western University, London, ON, Canada; ^6^Department of Surgery, Schulich School of Medicine and Dentistry, Western University, London, ON, Canada; ^7^Department of Medicine, Schulich School of Medicine and Dentistry, Western University, London, ON, Canada; ^8^Department of Biomedical Sciences, Ontario Veterinary College, University of Guelph, Guelph, ON, Canada; ^9^Department of Obstetrics and Gynaecology, Schulich School of Medicine and Dentistry, Western University, London, ON, Canada

**Keywords:** high-grade serous ovarian carcinoma, orthotopic models, inflammation, microenvironment

## Abstract

Ovarian high-grade serous carcinoma (HGSC) is an aggressive disease with an urgent need for improved therapies. Immunotherapies have proved useful for some cancers but have failed to provide benefits for HGSC. Improving our understanding of the mechanisms regulating the HGSC tumor microenvironment will facilitate the discovery of novel immunotherapies and help predict patient response. To this end, the development of syngeneic models is imperative to recapitulate immune responses observed in patients with HGSC. Yet, few syngeneic HGSC mouse models exist that accurately reflect the initiation and disease progression of human disease. In this study, we developed a syngeneic model reflecting both the site of origin and the genotype of early HGSC disease by deleting *Trp53* in mouse oviductal epithelial (OVE) cells. Orthotopic injection of OVE cells demonstrated advanced disease progression due to loss of *Trp53*, associated with a less active T cell phenotype. Molecular analyses uncovered altered inflammatory signaling in OVE4-*Trp53*ko cells. Further analysis on an ascites-derived cell line identified selection for decreased pro-inflammatory signaling. These results highlight potential mechanisms by which loss of p53 function contributes to an immunosuppressive microenvironment in HGSC, and provide insight into the role of ovarian and peritoneal microenvironments in regulating HGSC cell-intrinsic inflammatory signaling.

## INTRODUCTION

Ovarian high-grade serous carcinoma (HGSC) is a highly lethal disease with late-stage 5-year survival of 30% [1]. Most HGSC patients initially respond well to debulking surgery and chemotherapy, but effective treatment of frequent chemo-resistant recurrent disease remains elusive [2]. Immunotherapies that enhance the anti-tumor function of T cells, such as immune checkpoint inhibitors (ICIs) and adoptive T cell therapy, have yielded promising results for certain cancers [3, 4]. The efficacy of immunotherapy is largely determined by the inherent features of the tumor microenvironments (TMEs), including those of tumor-infiltrating of immune cells [5]. In ovarian cancer, the immunosuppressive environment and low mutation burden present challenges for immunotherapy [6, 7], and clinical trials of various immunotherapeutic regimens have yielded limited responses [8–12]. Improved understanding of the mechanisms regulating the HGSC immune microenvironment will help uncover new immunotherapeutic approaches and identify biomarkers to stratify patients for current immunotherapies.

The progression pattern of HGSC exposes premalignant and tumor cells to multiple unique microenvironments. Fallopian tube epithelial (FTE) cells are now recognized as the site of origin for the majority of HGSC cases [13–15]. The release of follicular fluid during ovulation bathes the fallopian tube with inflammatory cytokines and growths factors that promote disease progression [16]. Precursor lesions with universal *TP53* mutation exfoliate from the fallopian tube to form multicellular aggregates called spheroids that colonize the ovary, where interaction with the ovarian stroma further contributes to transformation [17]. Spheroids also mediate metastasis by passively spreading throughout the peritoneal cavity in ascites, a malignant fluid frequently accumulated in HGSC patients with both pro- and anti-inflammatory components [18, 19]. Within tumors, the immune microenvironment has prognostic value in HGSC, with infiltration of CD8+ T cells associated with favorable outcomes, and recruitment of regulatory T cells (Tregs) associated with reduced survival [20, 21]. Each of these environments presents distinct subsets of cells and signaling molecules with which malignant HGSC cells can interact. Identifying mechanisms by which common HGSC genomic alterations influence interactions with these microenvironments will improve our understanding of transformation events and help uncover immunotherapeutic targets. Since *TP53* mutation is universal in HGSC, identifying how this genomic alteration affects immune cell populations is particularly relevant.

Syngeneic mouse models are invaluable tools for research on immune responses to HGSC, as they can recapitulate human disease [22]. Several syngeneic HGSC models have been described; however, they are often derived from the ovarian surface epithelium or contain multiple genomic alterations [23–28]. While these studies, as well as studies in other cancer types, implicate loss of p53 function in contributing to an immunosuppressive phenotype [29, 30], whether this is reproducible in FTE cells with loss of p53 function alone remains unclear. In this study, we characterized the peritoneal and intratumoral T cell landscapes of a syngeneic, orthotopic model based on mouse oviductal epithelial cells (OVE; the murine equivalent to human FTE) with *Trp53* deletion only. *Trp53* deletion produced a less active T cell phenotype in the peritoneal cavity of mice. CD4+ T cell infiltration was reduced in OVE4-*Trp53*ko ovarian tumors compared to those of OVE4, largely driven by Foxp3+ Tregs. *In vitro* analyses indicated that the less active T cell phenotype was accompanied by reduced pro-inflammatory gene expression in OVE4-*Trp53*ko cells, which was further reduced in ascites-derived cells with decreased RelA signaling.

## RESULTS

### OVE4-*Trp53*ko cells produce advanced disease in a syngeneic, orthotopic mouse model of HGSC

**Figure 1:**
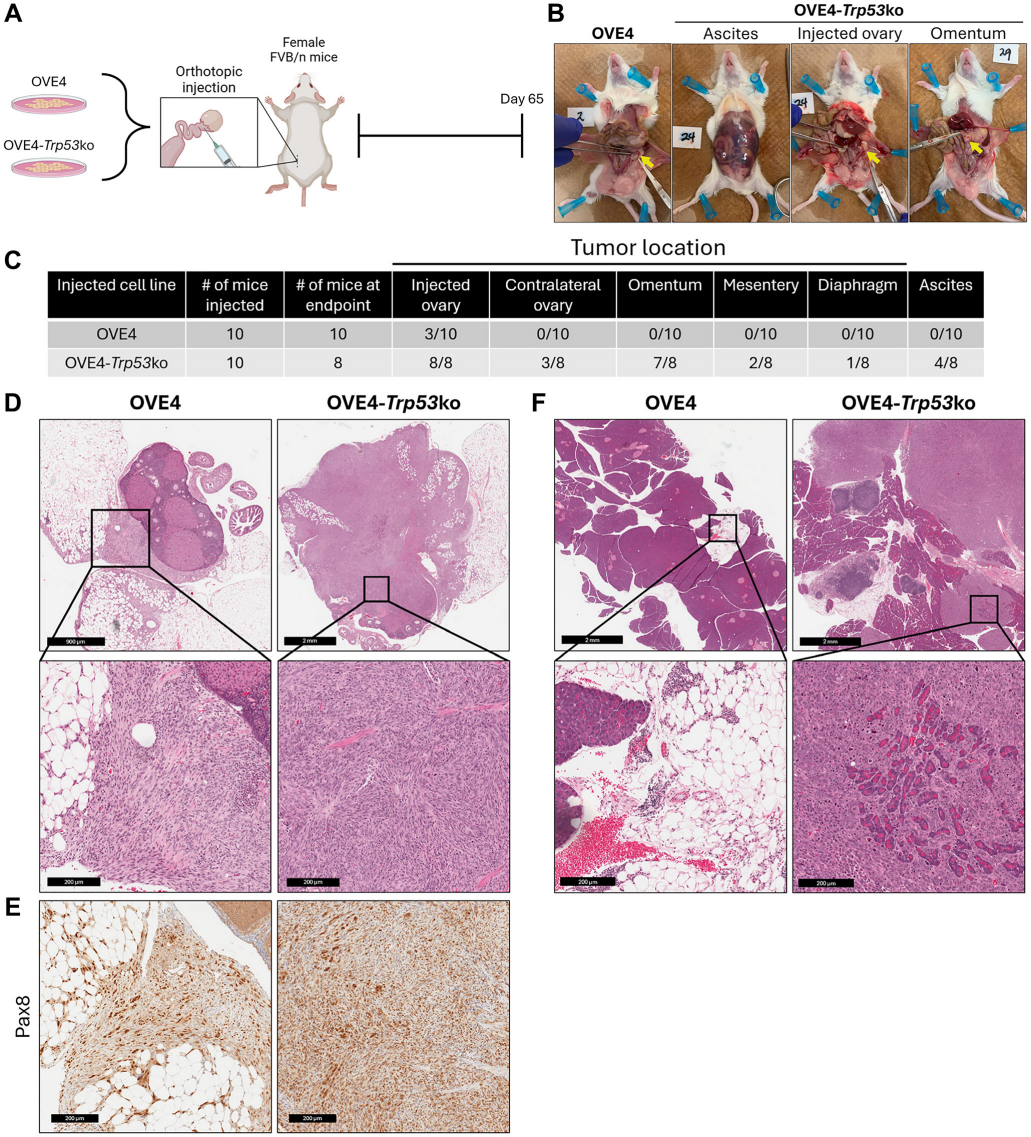
OVE4-*Trp53*ko cells produce advanced disease in a syngeneic, orthotopic mouse model of HGSC. (**A**) Schematic for orthotopic injection of OVE4 cell lines into FVB/n mice. (**B**) Representative gross anatomical images of OVE4 and OVE4-*Trp53*ko mouse groups with opened peritoneal cavity. In OVE4 images, injected ovaries are indicated by yellow arrow. In OVE4-*Trp53*ko images, injected ovary and omentum are indicated by yellow arrow. (**C**) Summary of disease burden for OVE4 and OVE4-*Trp53*ko mouse groups. (**D**) Representative images of H&E-stained injected ovary sections from OVE4 and OVE4-*Trp53*ko mouse groups. Insets represent high magnification images of regions containing OVE cells. (**E**) IHC for Pax8 in serial ovary sections from OVE4 and OVE4-*Trp53*ko mouse groups. (**F**) Representative images of H&E-stained omentum sections from OVE4 and OVE4-*Trp53*ko mouse groups. Insets represent high magnification images of normal omentum for OVE4 images, and high magnification images of regions containing OVE cells for OVE4-*Trp53*ko image.

Previously, we developed HGSC precursor cell lines by engineering p53 in the OVE4 and OVE16 lines [31]. *In vitro* characterization of these cell lines revealed a transformed phenotype due to mutation of the *Trp53* gene. To determine the effect of *Trp53* mutation on tumor formation in a mouse model accurately reflecting the microenvironment of HGSC initiation, OVE4 and OVE4-*Trp53*ko cell lines were injected into the oviducts of syngeneic female FVB/n mice (1 × 10^5^ cells; *n* = 10; Figure 1A). After 65 days, several mice injected with OVE4-*Trp53*ko cells showed signs of a moribund state, and all mice were sacrificed. Two mice injected with OVE4-*Trp53*ko cells were sacrificed before study endpoint due to extreme lethargy; one mouse had a large primary ovarian tumor, while the other mouse was sick from unknown causes. Both of these mice were excluded from subsequent analyses. Upon opening the abdominal cavity, tumor location was recorded (Figure 1B, 1C). In the OVE4-*Trp53*ko group, 8 mice that reached endpoint had large primary tumors at the injected ovary site, while few mice (3/10) in the OVE4 group had a small ovarian mass. Mice in the OVE4-*Trp53*ko group demonstrated widespread metastatic disease with tumors at the contralateral ovary (3/8), omentum (7/8), mesentery (2/8), and diaphragm (1/8), accompanied by ascites (4/8), while the OVE4 group had no evidence of metastatic disease. Histologically, tumor nodules within ovary tissues of the OVE4 group were in the ovary-adjacent fat pad, while OVE4-*Trp53*ko tumors invaded the ovary (Figure 1D). To confirm the OVE origin of these nodules, immunohistochemistry for the gynecologic epithelium marker Pax8 was performed (Figure 1E) [32, 33]. Omental tissues from the OVE4 group appeared histologically normal, while the omentum from mice injected with OVE4-*Trp53*ko cells frequently contained multiple large tumor nodules invading the adjacent pancreas (Figure 1F). Overall, when compared to the OVE4 cell line, the OVE4-*Trp53*ko cell line demonstrated increased tumor-forming potential and better reproduced invasion of the ovary as seen in human disease.

### Ascites-derived cell lines have enhanced survival and transformation properties compared to OVE4-*Trp53*ko

**Figure 2:**
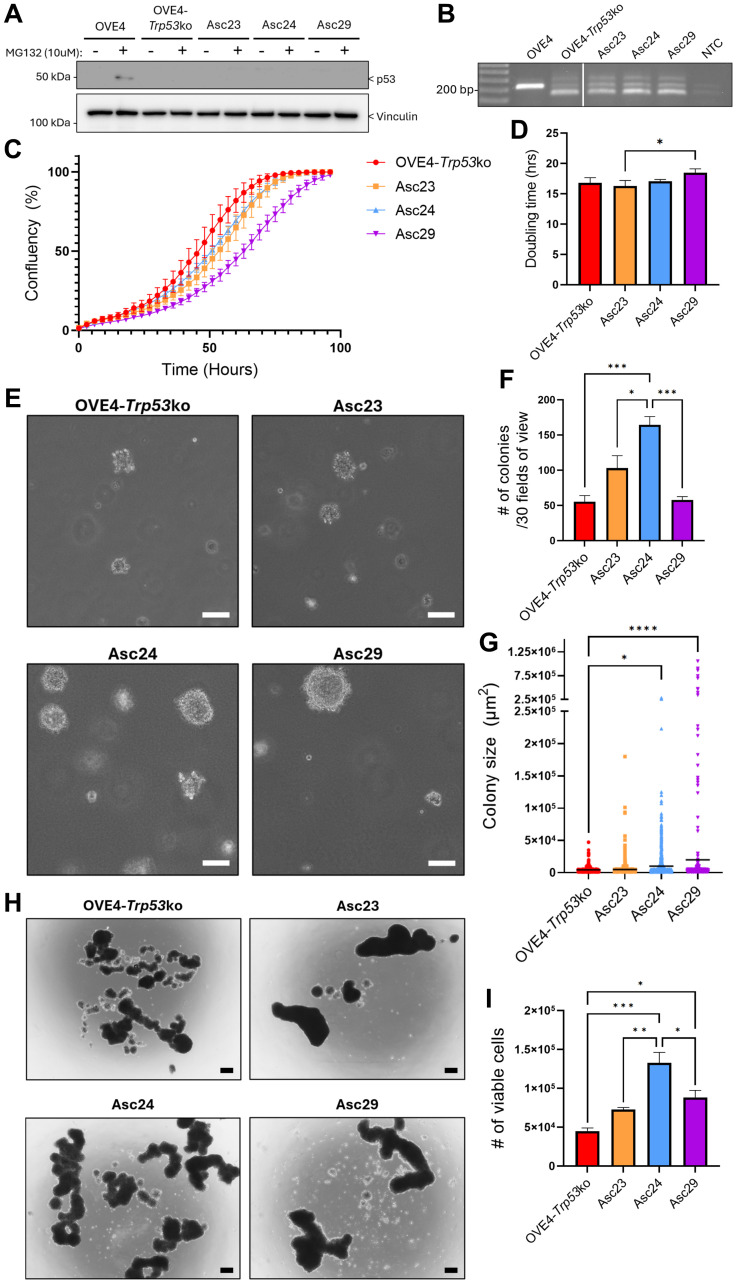
Ascites-derived cell lines have enhanced transformation properties. (**A**) Western blot analysis confirming lack of p53 protein expression in ascites-derived cell lines. Cells were treated with 10 μM of the proteasome inhibitor MG132 (or DMSO control) for 6 hours to detect p53. (**B**) PCR analysis confirming the OVE4-*Trp53*ko origin of ascites-derived cell lines using primers designed to amplify the endogenous mouse *Trp53* locus. (**C**) Growth curves and (**D**) doubling time of OVE4-*Trp53*ko and ascites-derived cell lines in adherent culture as determined by confluency-over-time data. Soft agar colony (**E**) morphology, (**F**) number, and (**G**) size for OVE4-*Trp53*ko and ascites-derived cell lines. Scale bars represent 100 μm. Spheroid (**H**) morphology and (**I**) cell viability of OVE4-*Trp53*ko and ascites-derived cell lines. Scale bars represent 200 μm. The number of viable cells was determined at day 3 by trypan blue exclusion counting. Statistical analyses were performed by one-way ANOVA followed by Tukey’s multiple comparisons test (^*^ *p* < 0.05; ^**^ *p* < 0.01; ^***^ *p* < 0.001; ^****^ *p* < 0.0001; *n* = 3). Error bars represent standard error of the mean.

The aggressiveness of the OVE4-*Trp53*ko cell line in a syngeneic, orthotopic mouse model points to pro-transformation changes in these cells due to deletion of *Trp53*. However, previous studies identified increased proliferation and expression of survival factors in a transformed mouse ovarian surface epithelium cell line due to interaction with the ovarian stroma [17]. Therefore, we asked whether phenotypic changes occurred in OVE4-*Trp53*ko cells following interactions with the ovarian microenvironment. Ascites-derived cell lines (Asc23, Asc24, Asc29) were established from three tumor-bearing mice orthotopically injected with OVE4-*Trp53*ko cells. Each of the ascites cell lines lacked detectable mouse p53 protein expression when treated with the proteasome inhibitor MG132 (Figure 2A). Additionally, deletion of the *Trp53* locus was confirmed by PCR as previously described (Figure 2B) [31].

To assess transformation properties in ascites-derived cell lines, proliferative capacity was assessed by measuring confluency-over-time (Figure 2C). Doubling times were similar among all cell lines except for Asc29, which exhibited a modest increase compared to Asc23 (Figure 2D). Next, anchorage-independent growth was measured by soft agar colony formation assays (Figure 2E). Asc24 formed the most colonies in soft agar (Figure 2F), and Asc24 and Asc29 had increased colony size compared to OVE4-*Trp53*ko (Figure 2G).

The ability of malignant cells to form spheroids following detachment and survive in suspension is a key determinant to both HGSC initiation and metastasis [34, 35]. Since the above-mentioned ascites-derived cell lines were established from mice with advanced disease, we postulated that they would have enhanced spheroid viability due to the selective pressures encountered following orthotopic injection. Indeed, when grown in suspension culture, ascites-derived cell lines formed robust spheroids of varying morphology (Figure 2H), accompanied by increased spheroid cell viability in Asc24 and Asc29 compared to OVE4-*Trp53*ko (Figure 2I). Overall, these results suggest that OVE4-*Trp53*ko cells acquire further pro-transformation changes following interaction with the host microenvironment.

### Close interaction with the ovarian microenvironment is not required for transformation of OVE4 cell lines

**Figure 3:**
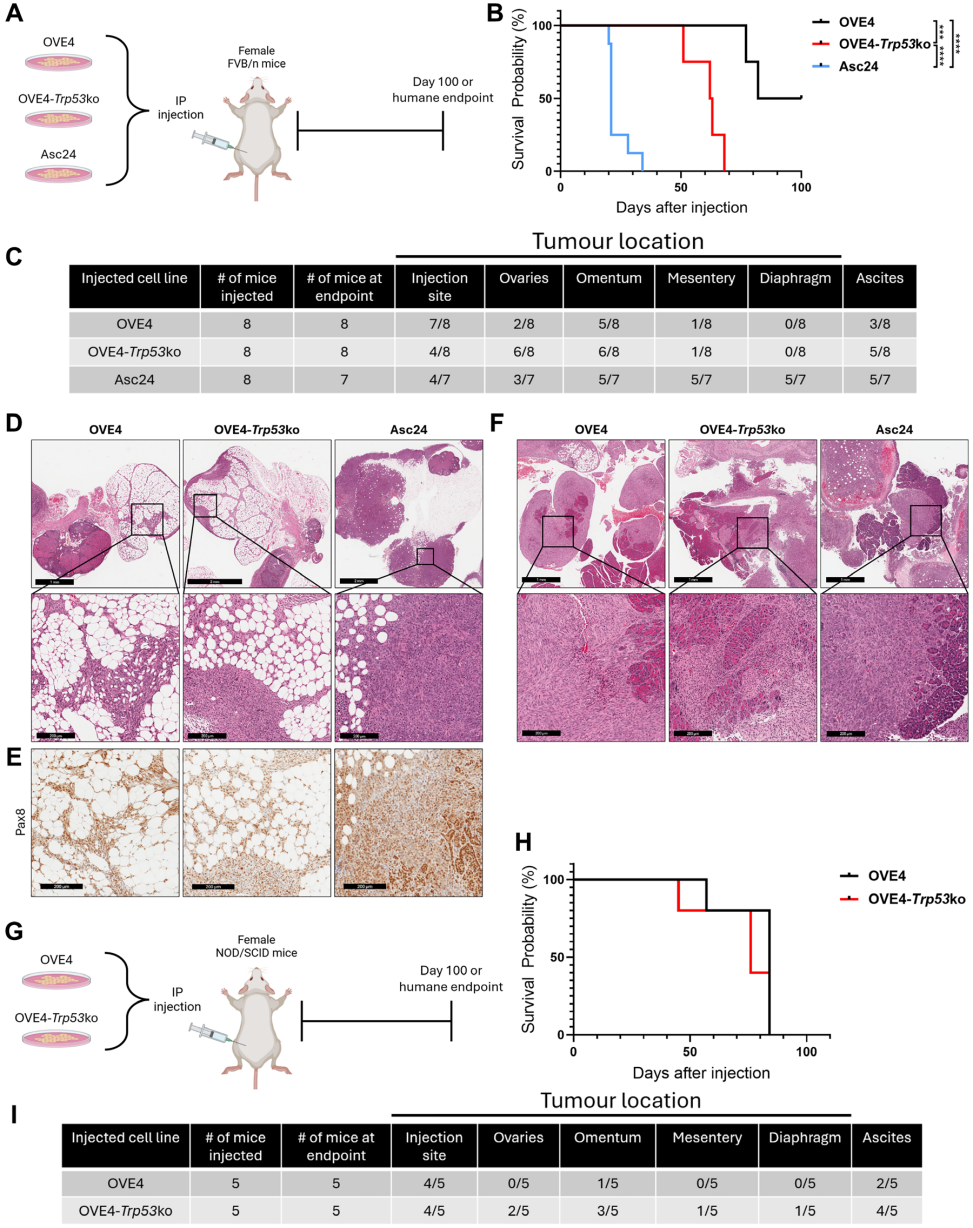
OVE4 and OVE4-*Trp53*ko cells are transformed in immune-competent and immune-compromised mouse models of HGSC. (**A**) Schematic for IP injection of OVE4 cell lines into FVB/n mice. (**B**) Survival curves for FVB/n mice injected IP with OVE4, OVE4-*Trp53*ko, and Asc24 cell lines. (**C**) Summary of disease burden for FVB/n mice injected IP with OVE4, OVE4-*Trp53*ko, and Asc24 cell lines. One of the mice in the Asc24 group was found deceased and necropsy could not be performed; it was excluded from this table. (**D**) Representative images of H&E-stained ovary sections from OVE4, OVE4-*Trp53*ko, and Asc24 FVB/n mouse groups. Insets represent high magnification images of regions containing OVE cells. (**E**) IHC for Pax8 in serial ovary sections from OVE4, OVE4-*Trp53*ko, and Asc24 FVB/n mouse groups. (**F**) Representative images of H&E-stained omentum sections from OVE4, OVE4-*Trp53*ko, and Asc24 FVB/n mouse groups. Insets represent high magnification images of regions containing OVE cells. (**G**) Schematic for IP injection of OVE4 cell lines into NOD/SCID mice. (**H**) Survival curves for NOD/SCID mice injected IP with OVE4 and OVE4-*Trp53*ko cell lines. (**I**) Summary of disease burden for NOD/SCID mice injected IP with OVE4 and OVE4-*Trp53*ko cell lines. Statistical analyses were performed by log-rank test for each comparison (^***^ *p* < 0.001; ^****^ *p* < 0.0001).

The hormone-rich ovarian microenvironment has been implicated in driving the transformation of fallopian tube epithelial cells [36]. Given the enhanced *in vivo* survival properties observed in the ascites-derived cell lines that had close interaction with the ovarian microenvironment, we asked whether OVE4 cell lines would produce similar disease progression when they are not confined to the oviductal space. To this end, OVE4 and OVE4-*Trp53*ko cell lines were injected directly into the peritoneal cavity of female FVB/n mice (4 × 10^6^ cells; Figure 3A). To address if the transformed phenotype in ascites-derived cell lines *in vitro* corresponds with increased tumor-forming and metastatic potential *in vivo*, the Asc24 cell line was included in this experiment. Mice injected with Asc24 cells had significantly reduced median survival (21 days) compared to mice injected with OVE4 cells (94 days) or OVE4-*Trp53*ko cells (62.5 days; Figure 3B). Tumor spread within each group is described in Figure 3C. Despite half of the mice in the OVE4 group surviving until study endpoint, all mice in this group formed tumors, with the injection site and omentum being the most common sites (7/8 and 5/8 mice, respectively). The OVE4-*Trp53*ko group had the highest frequency of ovarian tumors (6/8 mice) and exhibited tumors within their omentum (6/8 mice) and ascites accumulation (5/8 mice). Asc24 cells were the most metastatic, producing tumors within the omentum (5/7 mice), mesentery (5/7 mice), and diaphragm (5/7 mice), and producing ascites (5/7 mice).

Histology on ovarian tissues revealed that OVE4, OVE4-*Trp53*ko, and Asc24 all form tumor nodules in tissue surrounding the ovaries but fail to invade the ovary itself (Figure 3D). These peri-ovarian tumors contained high adiposity in the OVE4 and OVE4-*Trp53*ko groups, while those of the Asc24 group were densely packed with tumor cells. Immunohistochemistry for Pax8 confirmed the OVE origin of these tumors (Figure 3E). The omentum tumors in all three groups had similar histology, with densely packed tumor cells and invasion of the pancreas (Figure 3F). The disease progression produced by OVE4 cell lines in the intraperitoneal (IP) model indicates that close interaction with the ovarian microenvironment is not required for transformation. However, invasion of the ovary in the orthotopic model but not the IP model supports a pro-transformation role of the ovarian microenvironment and demonstrates increased accuracy of the orthotopic model with respect to human disease progression.

The disease progression observed in syngeneic orthotopic and IP models suggests the ability of OVE4 cell lines to overcome immune surveillance. To test whether the tumorigenicity of OVE4 and OVE4-*Trp53*ko cells is enhanced in the absence of a functional immune system, 4 × 10^6^ cells were injected into the peritoneal cavity of immune-compromised female NOD/SCID mice (Figure 3G). In contrast to the immune-competent model, there was no difference in median survival between the OVE4 (84 days) and OVE4-*Trp53*ko (76 days) groups in the immune-compromised NOD/SCID model (Figure 3H). However, the OVE4 group predominantly formed tumors at the injection site, while the OVE4-*Trp53*ko group had tumors across multiple sites (Figure 3I). Taken together, the results of these *in vivo* studies demonstrate increased tumor formation from OVE4-*Trp53*ko cells compared to OVE4 cells. Additionally, differences in disease spread and progression between the different models can highlight the involvement of ovarian and immune microenvironments in the transformation of OVE4 cell lines.

### OVE4-*Trp53*ko cells promote a less active T cell phenotype in the peritoneal cavity

HGSC frequently spreads from the primary ovarian tumor throughout the peritoneal cavity, and the resulting secondary lesions differ in their immune cell compositions [37, 38]. Additionally, the ascites fluid that mediates disease spread has a unique microenvironment itself, harboring a wide range of immune cells and cytokines [19]. Given the decreased survival of the OVE4-*Trp53*ko mouse group compared to the OVE4 mouse group observed in the syngeneic model but not the immune-compromised model, we sought to determine differences in immune cells following orthotopic injection of these two cell lines. As such, we enumerated classic immune cell types within ascites in the peritoneal cavity and those that infiltrated primary ovarian tumors. We focused on T cell populations and their functional phenotype, as their prognostic values in HGSC have been documented [20, 21, 39–41].

**Figure 4:**
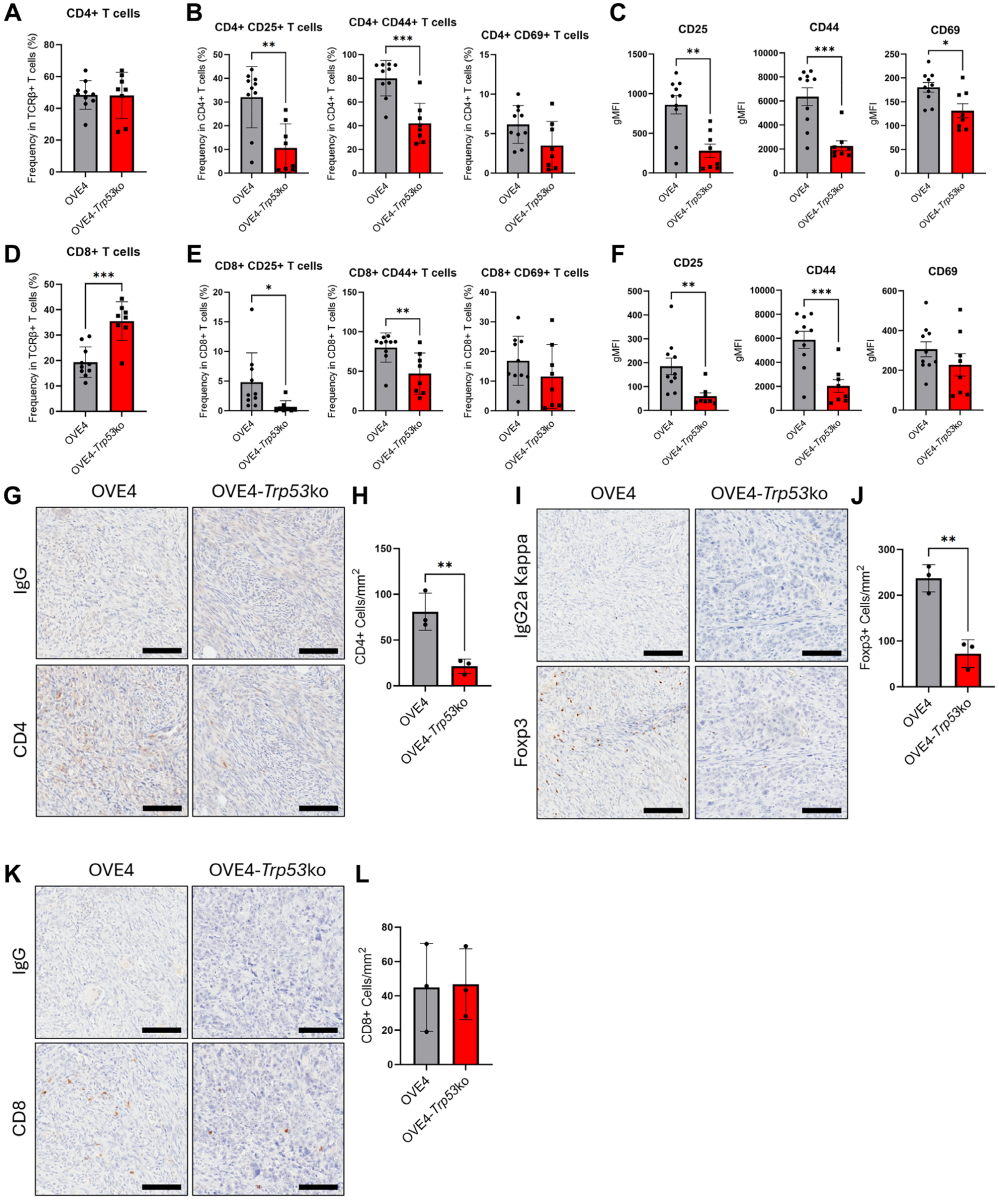
Mice injected with OVE4-*Trp53*ko cells have an altered T cell phenotype. (**A**) Frequency of peritoneal CD4+ T cells among TCRβ+ events. (**B**) Frequency of CD25+, CD44+, and CD69+ cells among peritoneal CD4+ T cells. (**C**) Geographic mean fluorescence intensity (gMFI) for CD25, CD44, and CD69 expression in peritoneal CD4+ T cells. (**D**) Frequency of peritoneal CD8+ T cells among TCRβ+ events. (**E**) Frequency of CD25+, CD44+, and CD69+ cells among peritoneal CD8+ T cells. (**F**) gMFI for CD25, CD44, and CD69 expression in peritoneal CD8+ T cells. Representative IHC images and the number of positive cells/mm^2^ for (**G**, **H**) CD4, (**I**, **J**) CD8, and (**K**, **L**) Foxp3 in sections of injected ovaries from OVE4 and OVE4-*Trp53*ko orthotopic injection mouse groups. Statistical analyses were performed by unpaired, two-tailed Student’s *t*-test (^*^ *p* < 0.05; ^**^ *p* < 0.01; ^***^ *p* < 0.001). Error bars represent standard deviation.

T cells in the peritoneal cavity were analyzed after collecting peritoneal wash samples at endpoint by flow cytometry with gating strategies shown in Supplementary Figure 1. The proportion of total CD4+ T cells was unchanged in the OVE4-*Trp53*ko group compared to the OVE4 group (Figure 4A), but CD25, CD44, and CD69 levels were reduced in CD4+ T cells of the OVE4-*Trp53*ko group (Figure 4B, 4C). Total CD8+ T cell frequencies were increased in the OVE4-*Trp53*ko group compared to the OVE4 group (Figure 4D). However, CD8+ T cells in the OVE4-*Trp53*ko group had decreased expression of activation markers CD25 and CD44 compared to the OVE4 group (Figure 4E, 4F).

Peritoneal immune cells represent an important immune cell population in HGSC, as they can directly interact with both metastasis-mediating spheroids that have exfoliated from solid tumors, as well as cells in the periphery of tumors throughout the peritoneal cavity. However, infiltrating immune cells have easier access to other cell types within the tumor bed. In our orthotopic model, only the OVE4-*Trp53*ko group had metastatic disease, which was accompanied by a less active T cell phenotype in the peritoneal cavity of OVE4-*Trp53*ko mice. Since both the OVE4 and OVE4-*Trp53*ko groups formed tumors at the ovary site, we asked whether the T cell phenotype within ovarian tumor tissues were similar between these groups. To quantify intratumoral T cells in ovarian tumor tissues, IHC for CD4, CD8, and the Treg marker Foxp3 was performed on tissue sections from OVE4 and OVE4-*Trp53*ko mouse groups. Ovarian tumors from the OVE4-*Trp53*ko group had fewer CD4+ cells (Figure 4G, 4H) and Foxp3+ cells (Figure 4I, 4J) compared to nodules of the OVE4 group. There was no difference in the proportion of intratumor CD8+ cells between the OVE4 and OVE4-*Trp53*ko groups (Figure 4K, 4L).

### OVE4-*Trp53*ko and ascites-derived cells have altered inflammatory signaling

Given that injection of OVE4-*Trp53*ko cells produced a less active T cell phenotype within the peritoneal cavity and ovarian tumors, molecular studies on tumor cell-intrinsic signaling pathways were warranted to investigate how OVE4-*Trp53*ko cells regulate the immune microenvironment. Previously, we identified inflammatory gene sets enriched in OVE4 spheroids compared to OVE4-*Trp53*ko spheroids [31]. Importantly, these analyses included an additional, independently isolated OVE cell line (OVE16) to provide an additional biological replicate. The identified inflammatory gene sets are associated with signaling pathways involving STAT and NFκB transcription factors, which regulate the expression of genes that mediate crosstalk between immune cells and non-immune cells. As such, we reassessed the RNA-seq data set with an emphasis on these regulatory pathways.

**Figure 5:**
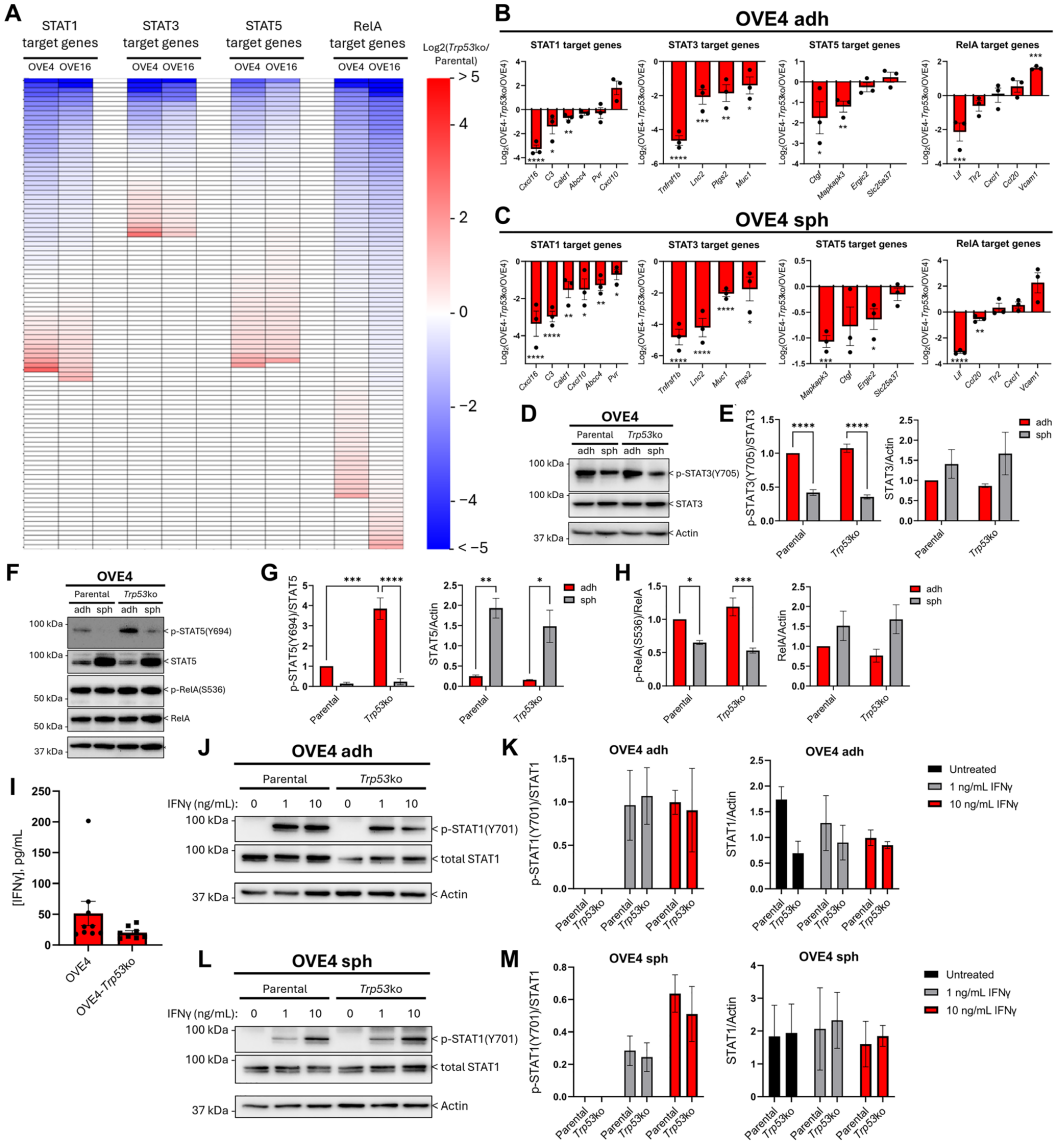
OVE4-*Trp53*ko cells have altered inflammatory signaling. (**A**) Log2(fold-change; *Trp53*ko/parental) heat maps for STAT1, STAT3, STAT5, and RelA transcription factor target gene expression from the OVE spheroid RNA-seq dataset [31]. See Supplementary Tables 3–10 for normalized read counts. RT-qPCR validation on cDNA from OVE4 and OVE4-*Trp53*ko (**B**) adherent and (**C**) spheroid cells for top hits within the STAT1, STAT3, STAT5, and RelA target gene lists. Representative western blots and densitometric analysis for (**D**, **E**) p-STAT3, (**F**, **G**) p-STAT5, and (F, **H**) p-RelA in OVE4 and OVE4-*Trp53*ko adherent and spheroid cells. (**I**) IFNγ concentrations in acellular peritoneal wash samples from orthotopic injection mouse groups as determined by ELISA. Representative western blots and densitometric analysis for IFNγ-induced p-STAT1 in OVE4 and OVE4-*Trp53*ko (**J**, **K**) adherent and (**L**, **M**) spheroid cells. Cells were treated with 0, 1, or 10 ng/mL IFNγ for 1 hour. Statistical analyses were performed by unpaired, two-tailed Student’s *t*-test for RT-qPCR data, and by one-way ANOVA followed by Tukey’s multiple comparisons test for densitometry data (^*^ *p* < 0.05; ^**^ *p* < 0.01; ^***^ *p* < 0.001; ^****^ *p* < 0.0001; *n* = 3). Error bars represent standard error of the mean for RT-qPCR data, and standard deviation for densitometry data.

First, we mined the RNA-seq data set and cross-referenced it with ChIP-seq-validated STAT1, STAT3, STAT5, and RelA target gene lists from the literature [42–45]. Target gene expression was summarized as a log_2_(fold-change; *Trp53*ko/parental) heatmap (Figure 5A). In agreement with our previous pathway analysis, most genes had lower expression in OVE spheroids with *Trp53* deletion compared to parental spheroids, with a small subset of genes that were lower in parental spheroids. To validate the RNA-seq data, transcript levels in OVE spheroids were measured for genes with the greatest decrease in expression due to *Trp53* deletion in both the OVE4 and OVE16 cell lines. We also measured transcript expression in adherent culture as differential regulation of inflammatory signaling in adherent culture compared to spheroid culture has been observed in epithelial ovarian cancer cells [46]. In adherent culture, expression of 3/6 STAT1 target genes, 4/4 STAT3 target genes, 2/4 STAT5 target genes, and 1/5 RelA target genes was decreased in OVE4-*Trp53*ko cells compared to OVE4 cells (Figure 5B). In spheroid culture, expression of 6/6 STAT1 target genes, 4/4 STAT3 target genes, 2/4 STAT5 target genes, and 2/5 RelA target genes was decreased in OVE4-*Trp53*ko cells compared to OVE4 cells (Figure 5C). Several inflammatory signaling molecules and receptors were among the genes decreased in OVE4-*Trp53*ko cells compared to OVE4 cells. The expression of STAT1 target genes *Cxcl16* and *C3*, STAT3 target gene *Tnfrsf1b*, and RelA target gene *Lif* was decreased in OVE4-*Trp53*ko cells in both adherent and spheroid culture, while the expression of STAT1 target gene *Cxcl10*, and RelA target gene *Ccl20* was decreased in spheroid culture only. Decreased expression of these inflammatory genes due to *Trp53* deletion was validated in OVE16 cells for *Cxcl10*, *Tnfrsf1b*, *Lif*, *Cxcl1*, and *Ccl20* in adherent culture, and *C3*, *Cxcl10*, *Tnfrsf1b*, *Cxcl1*, and *Trl2* in spheroid culture (Supplementary Figure 2A, 2B). Each of these genes have been associated with pro-inflammatory functions in cancer [47–53]. These results suggest that loss of p53 prevents pro-inflammatory signaling in OVE cells.

Since STAT1, STAT3, STAT5, and RelA target genes were decreased in OVE4-*Trp53*ko and OVE16-*Trp53*ko cells compared to parental controls, we next measured the activation status of these transcription factors. Despite the reduced expression of target genes in OVE4-*Trp53*ko cells compared to OVE4 cells, phosphorylation of STAT3 and RelA was unchanged between the two cell lines, and STAT5 phosphorylation was increased in adherent OVE4-*Trp53*ko cells compared to adherent OVE4 cells (Figure 5D–5H). STAT3 and STAT5 phosphorylation was similar in OVE16 and OVE16-*Trp53*ko cells, while p-RelA was decreased in adherent OVE16-*Trp53*ko cells compared to adherent OVE16 cells, in agreement with the target gene expression data for this transcription factor (Supplementary Figure 2C–2H). While there were limited changes in the activation of STAT3, STAT5, and RelA that were dependent on *Trp53* status, consistent culture condition-dependent changes in these transcription factors were observed in both the OVE4 and OVE16 cell lines. Except for p-STAT3 in the OVE16 cell lines, the phosphorylation of these transcription factors was decreased in spheroids of all cell lines compared to adherent culture, despite increased total protein in spheroid culture.

Phosphorylation of STAT1 at Tyr^701^ is commonly low or absent in untreated adherent cells but can be rapidly induced by treatment with interferon gamma (IFNγ) [54, 55]. IFNγ is frequently detected in the ascites of HGSC patients and is associated with improved outcomes [56, 57]. To address whether IFNγ-induced STAT1 signaling may be relevant in our orthotopic model, the presence of IFNγ in the peritoneal cavity of mice was confirmed by ELISA on acellular fractions of peritoneal wash samples (Figure 5I). When treated with 1 or 10 ng/mL IFNγ *in vitro*, all OVE cell lines had robust phosphorylation of STAT1 (Figure 5J–5M and Supplementary Figure 2I–2K). However, there were no significant differences in IFNγ-induced STAT1 phosphorylation between parental OVE cells and OVE cells with *Trp53* deletion.

### Interaction with the host microenvironment alters inflammatory signaling in OVE cells

**Figure 6:**
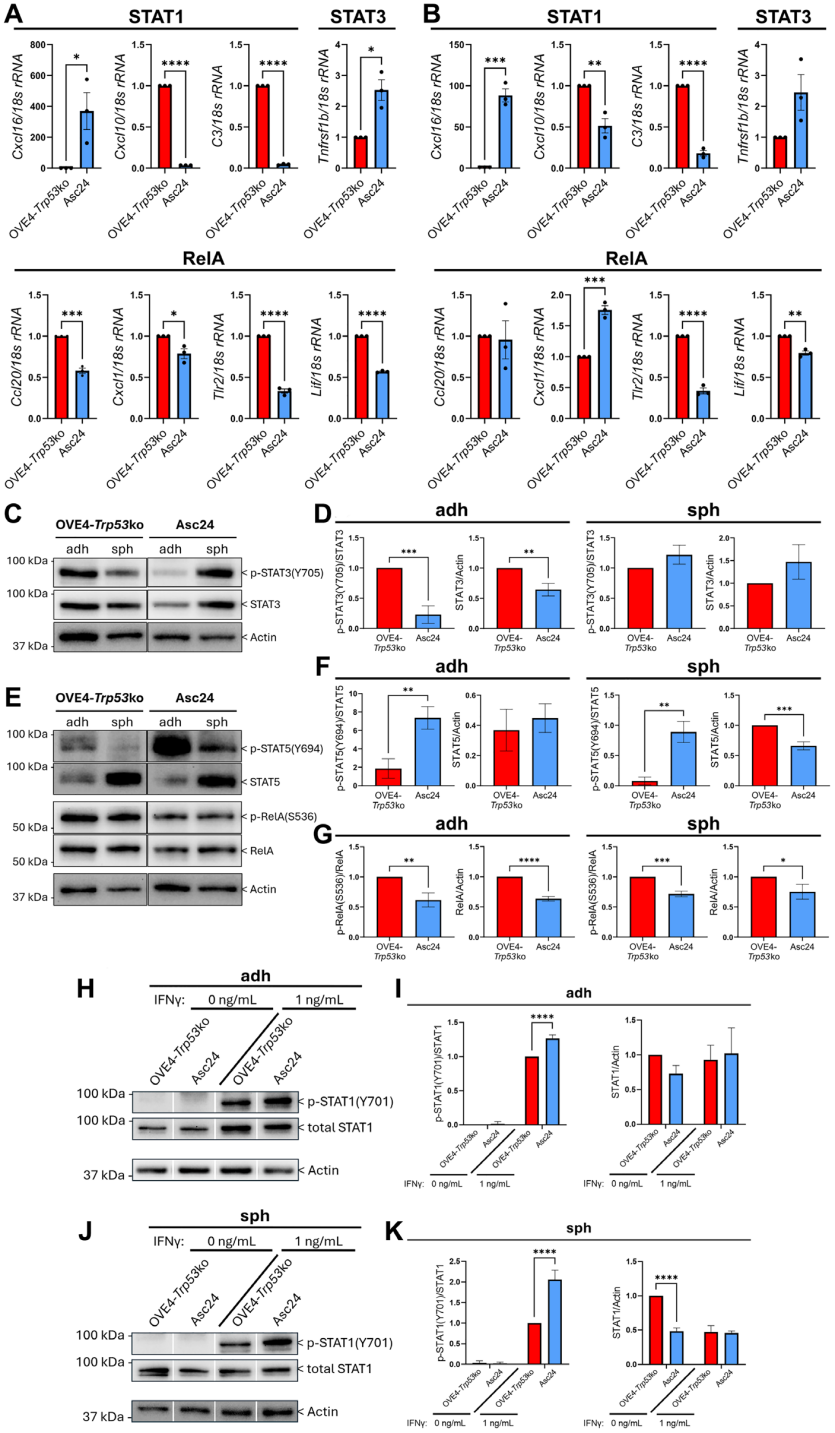
Ascites-derived cell lines have reduced pro-inflammatory signaling. RT-qPCR validation on cDNA from OVE4-*Trp53*ko and Asc24 (**A**) adherent and (**B**) spheroid cells for inflammatory target genes. Representative western blots and densitometric analysis for (**C**, **D**) p-STAT3, (**E**, **F**) p-STAT5, and (E, **G**) p-RelA in OVE4-*Trp53*ko and Asc24 adherent and spheroid cells. Representative western blots and densitometric analysis for IFNγ-induced p-STAT1 in OVE4-*Trp53*ko and Asc24 (**H**, **I**) adherent and (**J**, **K**) spheroid cells. Cells were treated with 0 or 1 ng/mL IFNγ for 1 hour. Statistical analyses were performed by unpaired, two-tailed Student’s *t*-test for RT-qPCR data and p-STAT3, p-STAT5, and p-RelA densitometry data. Statistical analyses were performed by one-way ANOVA followed by Tukey’s multiple comparisons test for p-STAT1 densitometry data (^*^ *p* < 0.05; ^**^ *p* < 0.01; ^***^ *p* < 0.001; ^****^ *p* < 0.0001; *n* = 3). Error bars represent standard error of the mean for RT-qPCR data, and standard deviation for densitometry data.

Despite the limited changes in STAT and RelA phosphorylation observed between OVE cell lines, the reduced expression of inflammatory genes in OVE cells with *Trp53*ko represents a mechanism by which OVE4-*Trp53*ko cells may alter T cell phenotypes in mice. However, increased anchorage-independent growth and spheroid viability in ascites cells compared to OVE4-*Trp53*ko cells demonstrates that the ascites-derived cell lines are not analogous to the originally injected OVE4-*Trp53*ko cells (Figure 2E–2I). As such, we proposed that interaction with the host microenvironment may have elicited changes in inflammatory signaling in the Asc24 cell line. Indeed, the expression of inflammatory target genes was significantly altered in Asc24 cells compared to OVE4-*Trp53*ko cells in both adherent and spheroid culture. In adherent culture, the expression of 6/8 pro-inflammatory genes was decreased in Asc24 compared to OVE4-*Trp53*ko, including 4/4 RelA target genes (Figure 6A). In spheroid culture, 4/8 pro-inflammatory genes were decreased in Asc24 (Figure 6B). Interestingly, *Cxcl16* followed an opposite trend to most of these pro-inflammatory genes, with increased expression in Asc24 cells compared to OVE4-*Trp53*ko cells in both adherent and spheroid culture.

To further assess the inflammatory signaling program in ascites-derived cells, STAT and RelA phosphorylation was measured in Asc24 and OVE4-*Trp53*ko cells. Asc24 cells had decreased p-STAT3 compared to OVE4-*Trp53*ko in adherent culture, partially driven by a decrease in total STAT3 protein (Figure 6C, 6D). STAT5 phosphorylation was increased in Asc24 compared to OVE4-*Trp53*ko in both adherent and spheroid culture (Figure 6E, 6F). RelA phosphorylation was decreased in Asc24 compared to OVE4-*Trp53*ko in both adherent and spheroid culture (Figure 6E, 6G), in agreement with the decreased RelA target gene expression observed in Asc24 cells. Since we confirmed the presence of IFNγ in the peritoneal cavity of all mice in the orthotopic injection study, we asked whether ascites-derived cell lines have altered IFNγ-induced STAT1 phosphorylation. Similar to the original OVE cell lines, Asc24 cells had no STAT1 phosphorylation in the absence of IFNγ (Figure 6H–6K). However, when treated with 1 ng/mL IFNγ, Asc24 cells had higher p-STAT1 compared to OVE4-*Trp53*ko cells in adherent and spheroid culture.

Overall, our *in vitro* molecular analyses highlight dampened pro-inflammatory signaling due to *Trp53* deletion that is further reduced following interaction with the ovarian microenvironment, likely driven in part by reduced RelA phosphorylation. These results support an OVE cell-intrinsic role in producing a pro-tumor immune microenvironment driven by *Trp53* deletion at multiple stages of disease progression.

## DISCUSSION

Immunotherapies represent a promising avenue to address the urgent need for improved HGSC treatment. To this end, the development of syngeneic models is of paramount importance. Most mouse models developed to date include mutation of *Trp53*, as this gene is universally mutated in HGSC. However, in many cases *Trp53* mutation is in the context of additional driver mutations, such as *PTEN* loss [22–24, 58, 59], activating *KRAS* mutations [23, 59], *BRCA* mutations [22–24, 59, 60], or *CCNE1* amplification [23, 59]. The limited studies assessing p53 mutation alone fail to recapitulate key aspects of HGSC tumorigenesis. For example, one group injected OVE cells with p53^R273H^ into the peritoneal cavity of immune-compromised mice and found no tumor formation [61]. This model lacks close interaction with the ovarian microenvironment and lacks an intact immune system. Another group found that *Trp53* deletion increased the tumorigenicity of the ID8 murine cell line model [25]. However, ID8 cells are derived from the ovarian surface epithelium. Additional syngeneic models derived from the cell of origin of HGSC with *Trp53* mutation only are required to study early transformation events.

We previously developed a novel HGSC precursor model and identified transformed properties due to *Trp53* mutation in OVE cells *in vitro* [31]. In this study, we applied our OVE model to an *in vivo* setting and discovered enhanced tumor-forming and metastatic potential in OVE4-*Trp53*ko cells. Of the HGSC transgenic mouse models with *Trp53* deletion or expression of a p53 missense mutant as the only genetic alteration, none have demonstrated tumor formation. For example, p53^R172H^ driven by the *amhr2* promoter or p53^R270H^ driven by the *Pax8* promoter are insufficient for disease progression on their own [62, 63]. However, these models both retain one wildtype *Trp53* allele. In another study, deletion of both *Trp53* alleles resulted in tumor formation only when mice were treated with high-ROS follicular fluid [64]. The discrepancy between these transgenic models and our result demonstrating advanced disease following injection of OVE4-*Trp53*ko cells may highlight the difference in the approach used for these models. Perhaps *Trp53* mutation within cells *in situ* is insufficient to promote their detachment from the oviduct and subsequent tumor growth, but *Trp53* loss can promote disease progression if cells are already detached, as is the case with our direct injection model.

Orthotopic injection provides the most accurate representation of the microenvironment in which HGSC initiates by placing cells within the oviduct, the site of origin for this disease. Recently, factors of this peri-ovarian microenvironment have been implicated in driving the initial transformation of FTE cells. Versican, a proteoglycan secreted by the ovary, increased FTE cell peritoneal metastasis *in vivo* [65]. Additionally, follicular fluid facilitated the transformation of human FTE cells and ovarian cancer cell lines [66], which may be dependent on loss of *TP53* [64]. When injected IP, cells are less exposed to these factors. In our IP model, all tumors at the ovary presented as nodules in the surrounding fat pad, while OVE4-*Trp53*ko cells could invade the ovary in the orthotopic model. This indicates that OVE cells injected IP are unable to invade through the ovarian bursa from the peritoneal cavity. However, OVE4-*Trp53*ko cells produced metastatic disease in the orthotopic model, suggesting that interaction with the ovarian microenvironment has enabled these cells to invade through the ovarian bursa from the oviductal space to the peritoneal cavity. This aligns with a previous study using OVE cells with an activating KRAS mutation and *Pten* loss, in which injection into the bursa produced advanced disease, while injection of the same number of cells into the peritoneal cavity produced no tumors [67]. IP studies using human HGSC cell lines have demonstrated a low frequency of ovarian invasion, despite the formation of extra-ovarian metastatic disease [68]. In one study, the ES-2, HEY, and OCC1 cell lines were incapable of invading the ovary, while the A2780-cp cell line formed ovarian tumors in only 12.5% of mice, which was associated with more aggressive disease [69]. Interestingly, when injected intravenous, human HGSC cell lines preferentially form tumors at the ovary [70]. Taken together, these human xenograft models suggest that aggressive HGSC cells can readily penetrate the ovaries of mice, but their ability to access the ovary may be hampered by the ovarian bursa.

In our IP model, parental OVE4 cells formed tumors in immune-competent and immune-compromised mice. While this cell line has no targeted HGSC driver mutations, spontaneous transformation of OVE cells *in vitro* has been observed [71]. Importantly, OVE4 cells retained increased expression of the canonical p53 target gene *Cdkn1a* and increased inferred p53 activity compared to OVE4-*Trp53*ko, indicating that OVE4 cells are an appropriate control with which to study *Trp53* mutation [31, 72]. Overall, orthotopic injection produces more aggressive disease, and better reproduces human disease progression compared to IP injection, supporting a pro-transformation role of the ovarian microenvironment.

Immune-compromised mice have been used for decades to circumvent human cancer cell xenograft rejection. When working with murine cell line models, injection into syngeneic, immune-competent mice is more common as this system more closely resembles the microenvironment of humans. In this study, we compared IP injection of OVE4 cell lines in immune-competent and immune-compromised models to address the how the host immune system affects disease progression. In the immune-competent model, half of the mice injected with OVE4 cells survived to study endpoint, while all mice injected with this cell line were euthanized by day 84 in the immune-compromised model. This supports the classical view on the immune system as a defense against cancer [73]. However, increasing evidence supports a pro-tumor role for certain aspects of the immune system. For example, conditioned media from macrophages increased migration and invasion of ovarian cancer cells [74, 75], and co-culture with CD8+ T cells increased the expression of metastasis-associated genes [76]. Indeed, the transformation of OVE4-*Trp53*ko may be enhanced by an intact immune system as injection of these cells resulted in a median survival of 62.5 days in immune-competent mice, but 72 days in immune-compromised mice. However, additional studies with larger sample sizes are required to validate these findings.

A recent study compared the immune profiles of multiple syngeneic HGSC models and found that FVB/n models were more immunogenic compared to C57BL/6 models due to high expression of MHC-I and MHC-II molecules [27]. Not surprisingly, the genotype of a cell line largely determines its immunogenicity in a syngeneic model, where cells with homologous recombination deficiency (HRD) promote the infiltration of intratumoral CD8+ T cells [23, 25]. In the current study, OVE4-*Trp53*ko cells promoted a less active CD8+ T cell phenotype, likely representative of cytotoxic T cells, in the peritoneal cavity of mice. With respect to CD4+ T cells, activation markers CD44 and CD69 were reduced in response to OVE4-*Trp53*ko cells. While CD25 represents an activation marker in CD8+ T cells and conventional CD4+ T cells, it is also expressed by Tregs. As such, reduced expression of CD25 in CD4+ T cells does not point to a functional outcome. Overall, decreased expression of CD44 and CD69 in CD4+ T cells, and CD25, CD44, and CD69 in CD8+ T cells in the OVE4-*Trp53*ko group suggests that loss of *Trp53* decreases the immunogenicity of OVE4 cells.

Reduced T cell infiltration and activity due to *Trp53* deletion has been observed in the C57BL/6 ID8 model [25, 27], and may be driven by hampered antigen presentation [77]. Interestingly, the frequency of CD4+ T cells was reduced due to *Trp53* deletion in ovarian tumors, but unchanged in the peritoneal cavity. In contrast, CD8+ T cells were unchanged in ovarian tumors, but increased due to *Trp53* deletion in the peritoneal cavity. Differences in T cell populations between ascites and solid tumors have previously been observed in humans and in mice [19, 23, 38]. Since T cell phenotypes are a key determinant of a patient’s response to immunotherapy [78], the unique T cell landscapes of tumors and ascites should be considered for prospective HGSC therapies. Due to the limited number of immune cells collected in peritoneal wash samples, our study only assessed T cell populations. Several other immune cell populations can influence HGSC disease progression including macrophages [79], myeloid-derived suppressor cells [80], natural killer cells [41], and neutrophils [81]. As such, future studies assessing these immune cells may uncover additional pro-tumor immune phenotypes in response to OVE4-*Trp53*ko cells.

Expression of inflammatory signaling molecules and receptors is one mechanism by which cancer cells can modulate the immune microenvironment. Reduced expression of pro-inflammatory genes due to *Trp53* deletion in OVE cells suggests a hampered ability to recruit immune cells. Surprisingly, STAT and RelA phosphorylation were not decreased in OVE cells with *Trp53* deletion, suggesting additional mechanisms of regulating inflammatory gene expression in these cells. One potential explanation is the involvement of other transcriptional cofactors that work in concert with STAT and RelA for efficient DNA binding and gene regulation [82–84]. There may be increased availability of these cofactors in parental OVE4 cells as compared with OVE4-*Trp53*ko. Alternatively, transcriptional activation in OVE4-*Trp53*ko cells may be hampered by members of the protein inhibitor of activated STATs (PIAS) family, which interact with STATs and RelA to repress their function [85, 86]. Importantly, further changes in gene expression were observed in ascites-derived cells. Altered expression of inflammatory genes due to treatment with follicular fluid has been observed in human FTE cells *in vitro*, implicating a role of the ovarian microenvironment in regulating inflammatory signaling [87]. The inflammatory phenotype of ascites-derived cell lines is likely more important in promoting disease progression compared to the inflammatory phenotype of OVE4-*Trp53*ko cells, as the ascites cells have undergone selection in an *in vivo* setting. We identified decreased expression in 4/4 RelA pro-inflammatory target genes in Asc24 cells compared to OVE4-*Trp53*ko cells in adherent culture, and decreased expression in 2/4 genes in spheroid culture. In agreement with this, upstream activation of RelA was also decreased in Asc24 cells compared to OVE4-*Trp53*ko cells. The function of RelA in ovarian cancer is generally considered to be pro-tumor through its role in anti-apoptotic gene activation, promotion of cancer cell stemness, and enhancing migration and invasion [88–90]. However, RelA also promotes the expression of pro-inflammatory genes in the context of other cancer types [91, 92]. Future studies assessing RelA signaling, and its regulation of inflammatory signaling and subsequent immune phenotypes at multiple time points, will address the multifaceted role of RelA in the OVE4 syngeneic model.

Expression of pro-inflammatory *Cxcl10* and *C3* was also decreased in Asc24 compared to OVE4-*Trp53*ko, independent of culture condition. The expression of *Cxcl10* and *C3* is likely driven by STAT1-idenpendent mechanisms, as STAT1 was not phosphorylated in Asc24 under basal conditions, and IFNγ-induced p-STAT1 was increased in Asc24 compared to OVE4-*Trp53*ko. Recently, Ishak et al. identified viral mimicry conditioning due to loss of p53 function in human FTE and in mouse ID8 cells, as well as in our OVE4 and OVE16 cell lines [93]. This increased tolerance to cytosolic DNA reduced the secretion of *Cxcl10* and decreased the proportion of T cells that were enriched for genes involved in non-self antigen recognition in the ID8 model. Several studies have identified a role of complement signaling in promoting an active T cell phenotype [94–96]. In ovarian cancer, high levels of complement protein are found in the ascites of patients, but malignant cells can acquire mechanisms to evade complement-mediated cytotoxicity [97]. A recent study identified *C3* as a novel p53 target gene in mouse embryonic fibroblasts [98], but whether p53 can activate *C3* in the context of ovarian cancer is not known. Our results support the association of functional complement signaling and T cell activation, as Asc24 cells with low *C3* expression produced an inactive T cell phenotype.

In summary, we identified an inactive T cell phenotype in response to orthotopic injection of OVE4-*Trp53*ko cells associated with decreased pro-inflammatory signaling. Analysis on ascites-derived cells identified a further reduction in the expression of these genes and reduced RelA activation following interaction with the ovarian microenvironment and subsequent selective pressure in an *in vivo* setting. The establishment of these ascites-derived cell lines provides tools for future studies. Additional altered inflammatory genes may be identified by transcriptomics, and the manipulation of specific genes of interest will provide further insight into the mechanisms by which HGSC cells modulate the immune microenvironment.

## MATERIALS AND METHODS

### Antibodies and reagents

For immunoblotting, antibodies against p53 (CAT# OP03; 1:1000), Vinculin (CAT# V9264; 1:50 000), and Actin (CAT# A2066; 1:20 000) were purchased from Sigma-Aldrich. Antibodies against p-STAT3-Y705 (CAT# 9131; 1:1000), STAT3 (CAT# 12640; 1:1000), p-STAT5-Y694 (CAT# 4322; 1:1000), STAT5 (CAT# 9363; 1:1000), p-RelA-S536 (CAT# 3033; 1:1000), RelA (CAT# 8242; 1:1000), p-STAT1-Y701 (CAT# 9167; 1:1000), and STAT1 (CAT# 9172; 1:1000) were purchased from Cell Signaling Technologies. HRP-conjugated antibodies against rabbit IgG (CAT# NA934; 1:10 000) and mouse IgG (CAT# NA931; 1:10 000) were purchased from Cytiva. Antibodies were diluted in tris-buffered saline-Tween 20 (TBST) containing 5% non-fat milk or 5% bovine serum albumin. For immunohistochemistry, antibodies against Pax8 (CAT# 10336-1-AP; 1:20 000) were purchased from Proteintech. Antibodies against CD4 (CAT# 25229; 1:200), CD8 (CAT# 98941; 1:500), and IgG isotype control antibodies (CAT# 3900; 1:200-1:500) were purchased from Cell Signaling Technologies. Antibodies against Foxp3 (CAT# 14-5773-82; 1:100) and IgG2a Kappa isotype control antibodies (CAT# 14-4321-81; 1:100) were purchased from Thermo Fisher Scientific. HRP-conjugated antibodies against rat IgG (CAT# 712-035-153; 1:1000) and rabbit IgG (CAT# 711-035-152; 1:1000) were purchased from Jackson ImmunoResearch. Antibodies for flow cytometry are described in Supplementary Table 1. Recombinant IFNγ was purchased from BioLegend (CAT# 575306). MG132 was purchased from Sigma-Aldrich (CAT# C2211).

### Cell lines

Parental OVE4 and OVE16 cell lines were provided by Dr. Barbara Vanderhyden [99]. *Trp53*ko and p53^R175H^-expressing derivatives were generated and maintained as previously described [31]. To generate ascites-derived cell lines, peritoneal wash samples were passed through sterile 70 μm cell strainers to separate OVE-derived multicellular spheroids from immune cells and peritoneal wash fluid. Ascites cells were plated in OVE growth media supplemented with 1× antibiotic-antimycotic (Gibco CAT# 15240062) for 2 weeks and expanded in standard OVE media for use. All cell lines were passaged at least 3 times after thawing prior to use and maintained at low passage number for *in vitro* and *in vivo* studies. Adherent cells were maintained on tissue culture-treated polystyrene plates (Sarstedt, unless otherwise specified). Spheroids were cultured in Ultra-Low Attachment plates (ULA; Corning).

### Animal studies

For orthotopic studies, female FVB/n mice (7–8 weeks old; Charles River Laboratories) were housed at the University of Guelph Central Animal Facility in accordance with the associated animal use protocol (#4668), and the guidelines of the Canadian Council on Animal Care. Mice were anesthetized with isoflurane, and the left ovary was accessed by a dorsal midline incision. OVE cells (1 × 10^5^ cells in 6 μL PBS) were injected through the ovarian bursa into the oviduct (*n* = 10 mice per group). All animals were euthanized at day 65, when several mice injected with OVE4-*Trp53*ko cells became moribund. For IP injections, female FVB/n mice and female NOD/SCID mice (7–8 weeks old; Charles River Laboratories) were housed in accordance with the associated animal use protocol (#2023-138), and the guidelines of the Animal Care Committee at Western University. OVE cells (4 × 10^6^ cells in 150 μL PBS) were injected in the peritoneal cavity (*n* = 8 mice per group for FVB/n; *n* = 5 mice per group for NOD/SCID). For survival analyses, weight and health scores were monitored twice a week, and mice were euthanized according to criteria for humane endpoint (extreme weight loss, visible tumor size, lethargy, abdominal bloating due to ascites, hunched posture, impaired breathing).

### Peritoneal wash

After euthanasia of mice in the orthotopic study, abdominal skin was incised to expose the peritoneal wall. Using a 10 mL syringe and 18-gauge needle, 5 mL PBS was injected into the peritoneal cavity. After massaging the abdomen to dislodge cells, PBS containing peritoneal cells was aspirated back into the syringe and transferred to a tube on ice. Peritoneal wash samples were strained through a 70 μm cell stainer to remove large cell clusters, which were cultured to generate ascites-derived cell lines. Remaining cells in PBS were centrifuged (1000 g; 5 min; 4°C) and resuspended in 1 mL PBS for flow cytometry. The supernatant was removed and stored at −80°C for ELISA.

### Histology and immunohistochemistry

Tissues collected at the time of necropsy were fixed in 10% formalin for 24 hours at 4°C, washed twice with PBS, and stored in 70% ethanol at 4°C. Fixed tissues were dehydrated through graded alcohols and embedded in paraffin. Embedded tissues were sectioned at 5 μm onto charged slides and stained with hematoxylin and eosin to visualize tissue structure. For immunohistochemistry, tissue sectioning and staining was performed by the Molecular Pathology Core Facility at Robarts Research Institute (London, ON, Canada). Tissue sections were imaged with the Aperio ScanScope slide scanner (Leica) and exported with ImageScope software package (Leica). The number of CD4+, CD8+, and Foxp3+ cells in tumor tissues was measured with the positive cell detection feature in the QuPath software package (version 0.5.1) [100].

### Preparation of whole cell lysates

Adherent cells at 80% confluency were washed twice with PBS and scraped into modified RIPA lysis buffer (RIPA buffer with 50 mM HEPES (pH 7.4), 150 mM NaCl, 10% glycerol, 1.5 mM MgCl_2_, 1 mM EGTA, 1% Triton X-100, 0.1% SDS, 1mM Na_3_VO_4_, 10 mM NaF, 1 mM PMSF, and 1x SIGMA*FAST* protease inhibitor cocktail (Sigma-Aldrich CAT# S8820)). Spheroid cells were pelleted to remove media, washed twice with PBS, and modified RIPA lysis buffer was added. Cells were incubated on ice for 30 min with vortexing every 5 min for complete lysis. Lysates were clarified by centrifugation (21 100 g; 20 min; 4°C), protein was collected from the supernatant and stored at −80°C.

### Immunoblot analysis

Protein lysates were loaded into wells of polyacrylamide gels (8 or 10%) and resolved by SDS-PAGE at 100 V for 1 hour 20 min. Proteins were transferred to PDVF membranes (Roche CAT# 03010040001) at 100 V for 1 hour. Non-specific binding was blocked with 5% non-fat milk or 5% bovine serum albumin in TBST for 1 hour at room temperature. Primary antibodies were diluted in blocking buffer and incubated on membranes overnight at 4°C. Membranes were washed with TBST (3 × 20 min), and HRP-conjugated secondary antibodies diluted in blocking buffer were incubated on membranes for 1 hour at room temperature. Membranes were exposed to chemiluminescent substrate and imaged in the ChemiDoc Imaging System (Bio-Rad) for visualization. Densitometry was performed with the Image Lab software package (version 6.1; Bio-Rad).

### PCR

DNA was isolated from adherent cells using the Wizard Genomic DNA Purification Kit (Promega CAT#A1120). The endogenous *Trp53* gene was amplified at exon 3 using primers described in Supplementary Table 2. Reactions were incubated in a MyCycler thermocycler (Bio-Rad) with the following cycle: 94°C for 5 min, (94°C for 45 sec, 60°C for 45 sec, 72°C for 45 sec) × 35, 72°C for 10 min. PCR products were run for 1 hour on a 1% agarose gel with the RedSafe nucleic acid stain (Intron Biotechnology CAT#21141) and imaged using the ChemiDoc Imaging System (Bio-Rad).

### Doubling time analysis

Cells were seeded into 48-well tissue culture-treated polystyrene plates (Corning CAT# 3548) at 7500 cells/well and imaged at 2-hour intervals in the IncuCyte S3 live cell analysis system (Sartorius). Confluency over time data was measured using the masking feature. Doubling times were calculated in GraphPad Prism by fitting an exponential growth equation to growth curve data.

### Growth in soft agar

Agarose A (Bio Basic CAT#D0012) was dissolved in diH_2_O at a concentration of 1% and autoclaved. Dissolved agar was diluted to 0.5% in media, and 1.5 mL was added to each well of a 6-well plate. After the agar solidified, trypsinized adherent cells were suspended in 1.5 mL 0.5% agar and added. After the agar with cells solidified, 2 mL of media was added. Fresh media was added every week, and colonies were imaged after 3 weeks. For quantification, 30 images of random fields of view were captured for each well. Images were exported to the Fiji image analysis software package [101], and colonies >1000 μm^2^ were counted and measured using the Trainable Weka Segmentation plugin (version 3.3.4) [102].

### Spheroid viability

Cells were seeded into 24-well ULA plates at 100 000 cells/well. At day 3, spheroids were pelleted to remove media, washed twice with PBS, and incubated in 50 μL Trypsin for 30 min at 37°C to dissociate into single cells. Trypsin was inactivated with 50 μL fetal bovine serum, and viable cells were counted by Trypan Blue exclusion in a TC20 cell counter (BioRad).

### Flow cytometry

Peritoneal cells were washed and resuspended in staining buffer (2% fetal bovine serum in PBS). Non-specific binding to Fcγ receptors was blocked by incubation with 5 μg/mL of an anti-CD16/CD32 monoclonal antibody (clone 2.4G2) for 15 min on ice. Cells were stained with primary antibodies against T cell surface proteins (Supplementary Table 1) for 30 min at 4°C, washed, and resuspended in staining buffer containing the 7-AAD viability dye to exclude dead cells. Stained cells were analyzed with a BD FACSCanto II flow cytometer, and data were analyzed with the FlowJo software package (version 10.8.1; BD Biosciences). The expression of activation and exhaustion markers was assessed after gating based on staining with isotype controls. The gating strategy is described in Supplementary Figure 1.

### RT-qPCR

Adherent cells at 80% confluency were washed twice with PBS, and pelleted. Spheroid cells were washed twice with PBS and pelleted. RNA was isolated with the RNEasy Spin Column Kit (Qiagen CAT# 74104) according to the manufacturer’s instructions. Genomic DNA contamination was removed by incubation with DNAseI (Qiagen CAT# 79254) for 30 min at 37°C. RNA concentration and purity was measured on a NanoDrop One Microvolume UV-VIS Spectrophotometer (Thermo Fisher Scientific). cDNA was generated from 2 μg RNA with the High-Capacity cDNA Reverse Transcription Kit (Thermo Fisher Scientific CAT# 4368814), with a final volume of 40 μL/reaction. Reactions were incubated in a MyCycler thermocycler (Bio-Rad) with the following cycle: 25°C for 10 min, 37°C for 120 min, 85°C for 5 min. cDNA was diluted 1:2 in nuclease free H_2_O. qPCR was performed with the GB-Amp InFluor Green qPCR Mix (GeneBio Systems CAT# P2092) according to the manufacturer’s instructions, in a QuantStudio 3 RT-PCR system (Thermo Fisher Scientific) with the following cycle: 50°C for 2 min, 95°C for 10 min, (95°C for 15 sec, 60°C for 1 min) × 40, 95°C for 15 sec, 60°C for 1 min, 95°C. Fold-change expression of target genes relative to the *18s rRNA* housekeeping gene control was performed using the QuantStudio Design and Analysis Software package (version 1.1.0) using the 2^−ΔΔC^_T_ method. Primers were purchased from Invitrogen (Supplementary Table 2).

### ELISA

IFNγ in acellular peritoneal wash samples was analyzed using the Mouse IFNγ ELISA Kit (Thermo Fisher Scientific CAT# KMC4021) according to the manufacturer’s instructions, and measured with a BioTek Synergy H1 plate reader (Agilent Technologies).

### Statistical analyses

Statistical analyses were performed with GraphPad Prism (version 9.4.1). Specific statistical analyses performed for each experiment are described in figure legends.

## SUPPLEMENTARY MATERIALS



## Abbreviations

HGSC: high-grade serous carcinoma
OVE: oviductal epithelial
ICI: immune checkpoint inhibitors
TME: tumor microenvironment
FTE: fallopian tube epithelium
Treg: regulatory T cell
IFNγ: interferon gamma
IP: intraperitoneal
