# Supplementary material for: Loss of Trp53 results in a hypoactive T cell phenotype accompanied by reduced pro-inflammatory signaling in a syngeneic orthotopic mouse model of ovarian high-grade serous carcinoma
Source: Oncotarget. 2025 Sep 22;16:697–718. doi: 10.18632/oncotarget.28768 (PMC12453223; doi:10.18632/oncotarget.28768)
Supplement: Supplementary file 1 [file oncotarget-16-28768-s001.pdf]

Loss of *Trp53* results in a hypoactive T cell phenotype accompanied by reduced pro-inflammatory signaling in a syngeneic orthotopic mouse model of ovarian high-grade serous carcinoma

SUPPLEMENTARY MATERIALS

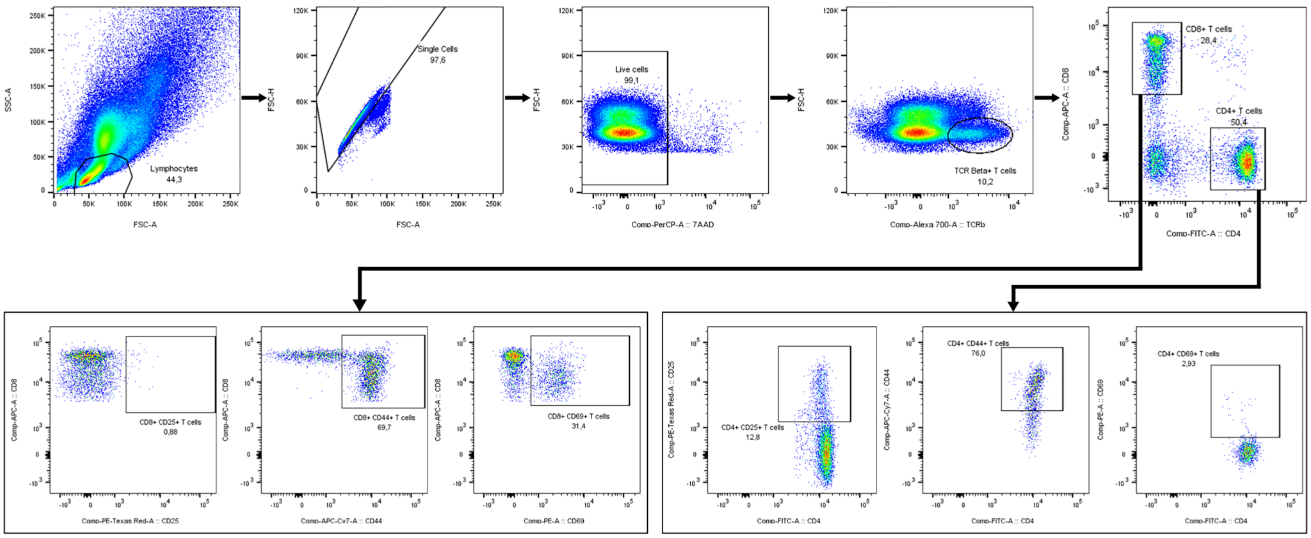

Supplementary Figure 1: Flow cytometry gating strategy for identifying CD4+ TCRβ+ T cells, CD8+ TCRβ+ T cells, and the expression of CD25, CD44, and CD69 within CD4+ and CD8+ T cells.

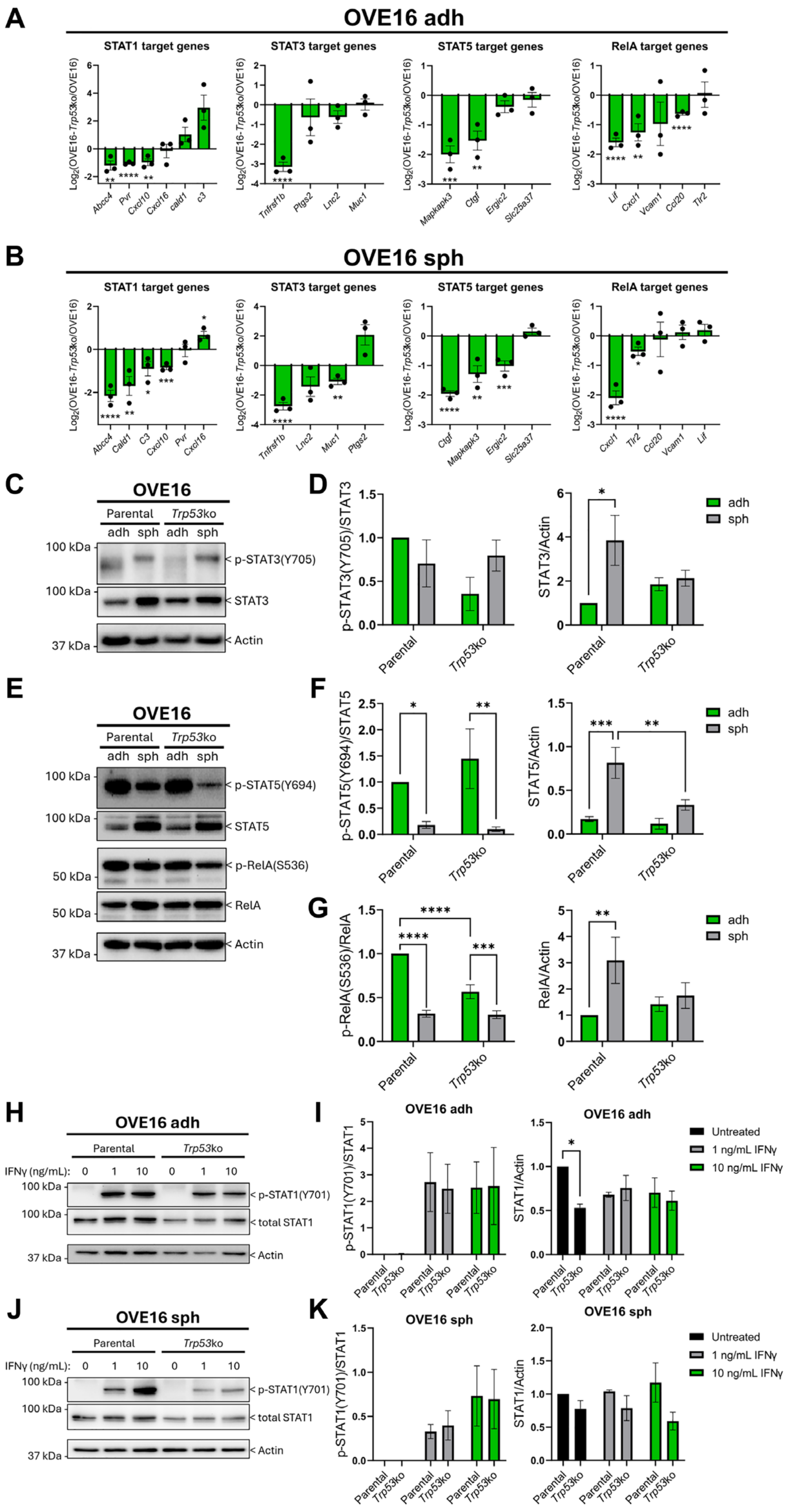

Supplementary Figure 2: Inflammatory signaling in OVE16 cell lines. RT-qPCR validation on cDNA from OVE16 and OVE16-*Trp53*ko (**A**) adherent and (**B**) spheroid cells for top hits within the STAT1, STAT3, STAT5, and RelA target gene lists. Representative western blots and densitometric analysis for (**C**, **D**) p-STAT3, (**E**, **F**) p-STAT5, and (E, **G**) p-RelA in OVE16 and OVE16-*Trp53*ko adherent and spheroid cells. Representative western blots and densitometric analysis for IFNγ-induced p-STAT1 in OVE16 and OVE16-*Trp53*ko (**H**, **I**) adherent and (**J**, **K**) spheroid cells. Cells were treated with 0, 1, or 10 ng/mL IFNγ for 1 hour. Statistical analyses were performed by unpaired, two-tailed Student's *t*-test for RT-qPCR data, and by one-way ANOVA followed by Tukey's multiple comparisons test for densitometry data ($^{*}p < 0.05$; $^{**}p < 0.01$; $^{***}p < 0.001$; $^{****}p < 0.0001$; $n = 3$). Error bars represent standard error of the mean for RT-qPCR data, and standard deviation for densitometry data.

Supplementary Table 1: Antibodies used for flow cytometry staining of peritoneal immune cells

Target	Clone	Fluorochrome	CAT#	RRID
TCR-beta	H57-597	AF700	Thermo Fisher Scientific CAT# 56-5961-82	AB_2802349
CD4	RM4-5	FITC	Thermo Fisher Scientific CAT# 11-0042-81	AB_464895
CD8a	53-6.7	APC	Thermo Fisher Scientific CAT# 17-0081-81	AB_469334
CD25	PC61.5	PE-eFluor610	Thermo Fisher Scientific CAT# 61-0251-80	AB_2574541
CD44	IM7	APC-eFluor780	Thermo Fisher Scientific CAT# 47-0441-80	AB_1272248
CD69	H1.2F3	PE	Thermo Fisher Scientific CAT# 12-0691-81	AB_465731
CD25 isotype control (Rat IgG1κ)	eBRG1	PE-eFluor610	Thermo Fisher Scientific CAT# 61-4301-82	AB_2637348
CD44 isotype control (Rat IgG2bκ)	eB149/10H5	APC-eFluor780	Thermo Fisher Scientific CAT# 47-4031-80	AB_1272021
CD69 isotype control (AH IgG)	eBio299Arm	PE	Thermo Fisher Scientific CAT# 12-4888-81	AB_470073
PD-1	J43	PE-eFluor610	Thermo Fisher Scientific CAT# 61-9985-80	AB_2574687
LAG-3	eBioC9B7W	PE-Cy7	Thermo Fisher Scientific CAT# 25-2231-80	AB_2573427
TIM-3	RMT3-23	PE	Thermo Fisher Scientific CAT# 12-5870-81	AB_465973
PD-1 isotype control (AH IgG)	eBio299Arm	PE-eFluor610	Thermo Fisher Scientific CAT# 61-4888-82	AB_2637314
LAG-3 isotype control (Rat IgG1κ)	eBRG1	PE-Cy7	Thermo Fisher Scientific CAT# 25-4301-81	AB_470197
TIM-3 isotype control (Rat IgG2aκ)	eBR2a	PE	Thermo Fisher Scientific CAT# 12-4321-80	AB_1834380

Supplementary Table 2: Primers for RT-qPCR and PCR

Primers for RT-qPCR		
**Gene**	**Primer Direction**	**Sequence (5' to 3')**
Cxcl16	Forward	CGTGAACTAGTGGACTGCTTT
Cxcl16	Reverse	GTGCTCGTGTCCGAAGGT
C3	Forward	CGCAACGAACAGGTGGAGATCA
C3	Reverse	CTGGAAGTAGCGATTCTTGGCG
Cald1	Forward	CTGTCAGAGGACAAGAAGCCGT
Cald1	Reverse	GGAGACTACTGCTGCTTGGTGA
Cxcl10	Forward	GTGTTGAGATCATTGCCACG
Cxcl10	Reverse	TCACTCCAGTTAAGGAGCCC
Abcc4	Forward	CACTCAGGAAACGAACCTTCTCC
Abcc4	Reverse	TTGCACTGCCTGCGTGTTCTCT
Pvr	Forward	AACTGGAGCACGAACACGG
Pvr	Reverse	GACTTCGCACACAATGACCG
Tnfrsf1b	Forward	TGACAGGAAGGCTCAGATGTGC
Tnfrsf1b	Reverse	ATGCTTGCCTCACAGTCCGCAC
Lcn2	Forward	ATGTCACCTCCATCCTGGTCAG
Lcn2	Reverse	GCCACTTGCACATTGTAGCTCTG

Muc1	Forward	AGTGCCTCTGACGTGAAGTCAC
Muc1	Reverse	GGGAGGGAACTGCATCTCATTC
Ptgs2	Forward	GCGACATACTCAAGCAGGAGCA
Ptgs2	Reverse	AGTGGTAACCGCTCAGGTGTTG
Mapkapk3	Forward	GTGGATTCCCACCCTTCTACTC
Mapkapk3	Reverse	GTCTTCAGGAGCAGGCGGATTA
Ctgf	Forward	TGCGAAGCTGACCTGGAGGAAA
Ctgf	Reverse	CCGCAGAACTTAGCCCTGTATG
Ergic2	Forward	TCACTGACTCCAGATGCGTGCA
Ergic2	Reverse	TGGTTGACAAGTGCTGCCAAATG
Slc25a37	Forward	GCCACCCTACTCCACGATGCA
Slc25a37	Reverse	CAACTGAAGGCTGACTGGTGCT
Lif	Forward	TCAACTGGCACAGCTCAATGGC
Lif	Reverse	GGAAGTCTGTCATGTTAGGCGC
Ccl20	Forward	TCTTCCTTGCTTTGGCATGGG
Ccl20	Reverse	GTCGTAGTTGCTTGCTGCTTCTG
Tlr2	Forward	ACAGCAAGGTCTTCCTGGTTCC
Tlr2	Reverse	GCTCCCTTACAGGCTGAGTTCT
Cxcl1	Forward	GTCCAAAAGATGCTAAAAGGTGTCC
Cxcl1	Reverse	AGAAGCCAGCGTTCACCAGA
Vcam1	Forward	GCTATGAGGATGGAAGACTCTGG
Vcam1	Reverse	ACTTGTGCAGCCACCTGAGATC
18s rRNA	Forward	CCATCCAATCGGTAGTAGCG
18s rRNA	Reverse	GTAACCCGTTGAACCCCATT

Primers for PCR

Gene	Primer Direction	Sequence (5' to 3')
Trp53	Forward	TTTGGTGTTGGGCTGGTAGG
Trp53	Reverse	CATGGAGTGGCTGGGGC

Supplementary Table 3: RelA target gene expression in OVE4 and OVE4-*Trp53*ko from spheroid RNA-seq data

RelA target genes	Normalized read counts						
Gene symbol	OVE4 (1)	OVE4 (2)	OVE4 (3)	OVE4-*Trp53*ko (1)	OVE4-*Trp53*ko (2)	OVE4-*Trp53*ko (3)	Log2(OVE4-*Trp53*ko/OVE4)
Cxcl16	300.3582086	270.9222574	163.1591624	4.49308453	2.751279177	2.051660216	−6.303886313
Icam1	2700.250033	2517.983334	2305.461315	104.4642153	77.03581694	124.1254431	−4.621604595
Lif	368.7566125	203.8207695	354.2565375	35.94467624	40.35209459	47.18818496	−2.907975555
Rrad	455.9893595	454.6125806	293.909998	68.51953908	95.37767812	67.70478712	−2.378729049
Atp2b4	91.19787191	36.90581835	13.41034211	2.246542265	16.50767506	11.28413119	−2.236067714
Tnfaip2	5798.995116	5309.405231	4190.731911	1355.788257	1044.568994	1182.782114	−2.094153765
Csf1	24600.62595	27848.79502	20020.52325	6139.80001	5741.002548	7383.925117	−1.911420992
Cxcl10	25.77331163	13.42029758	6.705171057	5.616355663	1.834186118	5.129150539	−1.867359179
Birc3	954.6038114	853.027665	696.2202614	288.6806811	210.9314035	239.0184151	−1.761224397

Ccl20	113.9973399	15.93660338	41.34855485	14.60252472	6.419651412	30.77490324	−1.725435085
Bcl3	308.2884583	336.3462081	308.4378686	99.97113079	76.11872388	132.3320839	−1.627680627
Rhbdf2	454.0067971	413.5129192	421.3082481	142.6554338	161.4083784	185.6752495	−1.395974527
Pdgfb	1188.546178	1200.277865	1084.002654	329.1184418	375.0910611	620.6272153	−1.390295855
Cxcl1	4484.556223	1611.274478	1663.999951	1255.817126	583.2711854	1241.254431	−1.332934283
C3h12a	675.0625084	612.3010772	640.3438359	232.5171244	186.1698909	375.4538195	−1.279419317
Gbp2	1367.968079	1607.080635	1347.739382	605.4431404	447.5414127	793.9925035	−1.226796172
Tlr2	824.745972	750.697896	645.9314785	370.6794737	242.1125675	342.627256	−1.217247265
Tnfaip3	3863.0229	2662.251533	2848.580171	1682.660156	1119.770625	1404.361418	−1.155921862
Pdlim4	1006.150435	1038.395525	1038.183985	511.0883653	467.71746	437.003626	−1.1225814
Map3k8	55.51174812	44.45473574	41.34855485	19.09560925	11.00511671	35.90405378	−1.098272599
Cxcl5	1540.45101	1178.469881	1960.145006	1114.284963	409.9405973	973.5127724	−0.905598201
Alpk1	586.8384801	596.3644738	388.8999213	240.3800224	295.3039649	322.1106539	−0.873991419
Rnd1	559.082606	305.31177	186.6272611	197.6957193	241.1954745	155.9261764	−0.821273686
Ell2	1669.317568	1664.955669	1874.09531	1138.996928	899.6682907	1104.819026	−0.728467006
Itpkc	722.6440068	724.6960694	731.9811737	521.1978055	335.6560595	463.6752088	−0.722762812
Nfkbiz	4584.675626	4075.576622	5222.210725	2377.964988	2233.121598	3824.294642	−0.718738372
Mt2	2122.333084	1524.042544	1232.633946	1117.654777	643.7993273	1322.295009	−0.661902919
Psmb10	86.24146583	119.105141	113.987908	64.02645455	63.27942106	76.93725809	−0.644780915
Cxcl2	346.9484257	84.71562848	111.7528509	157.2579586	75.20163082	122.0737828	−0.616138012
Serpina3g	260.7069599	249.1142739	415.7206055	180.8466523	159.5741922	274.9224689	−0.588906663
Camkk2	1009.124278	1002.328476	748.7441013	575.1148198	669.477933	640.1179873	−0.550428043
Rab20	729.5829753	868.1254998	1043.771628	630.1551053	555.7583937	660.6345895	−0.516515709
Rnf19b	682.9927581	774.1834167	692.8676759	585.22426	451.2097849	507.7859034	−0.477487829
Traf3	1279.74405	1203.632939	1046.006685	773.9338103	890.4973601	890.4205336	−0.466176719
Vcam1	2896.523714	2054.983067	1162.22965	1301.871243	1355.463541	1776.737747	−0.463421988
Tapbp	3478.405788	4539.415657	3968.343737	2894.669708	2860.413251	3111.342717	−0.434945628
Nfkbib	284.4977091	272.5997946	319.6131537	196.5724482	250.3664051	223.6309635	−0.386713239
Klhl25	294.4105213	321.2483734	258.1490857	251.6127337	227.4390786	194.9077205	−0.374654313
Rsl1	81.28505974	104.8460749	86.04969523	70.76608135	66.9477933	75.91142798	−0.349482923
Nfkbie	210.1516179	134.2029758	100.5775659	128.0529091	98.1289573	127.2029334	−0.332345394
Nfkb2	1712.933942	1653.212908	1468.432461	1225.488806	1405.903659	1356.147403	−0.277891413
Gch1	1865.591249	1787.415884	1936.676907	1996.052802	1406.820752	1313.062538	−0.245222568
Gem	3207.786016	3318.168577	2746.885076	2735.165208	2539.43068	2623.047586	−0.231588969
Clic4	5989.321109	7006.234106	6895.150903	5223.210766	5819.872551	5947.762965	−0.22733678
Sqstm1	23185.07637	23990.45946	22699.23908	21706.09136	18691.27363	20388.37339	−0.201038854
Rab8b	1010.11556	1020.781385	1071.709841	1039.025798	802.4564265	864.7747809	−0.197182012
Nfkbia	8872.958168	7419.747025	8067.43831	7456.273778	6485.682112	7367.511835	−0.193028075
Pim1	1100.32215	891.6110206	886.200108	894.1238215	857.48201	813.4832755	−0.166124457
Fas	458.9632032	327.9585221	421.3082481	396.5147098	286.1330344	394.9445915	−0.165083587
Uap1	3167.143486	3240.163097	2975.978421	3211.432168	2758.615921	2416.855734	−0.161954774
Noct	579.8995116	480.6144071	505.1228863	454.9248087	537.4165325	461.6235485	−0.106757145
C9orf72	856.466971	968.7777317	879.494937	830.0973669	795.119682	903.756325	−0.096937878

Rel	522.405201	526.7466801	470.4795025	459.4178932	502.5669962	459.5718883	−0.09624969
Zswim4	938.7433119	657.5945815	813.5607549	777.3036237	719.0009581	773.4759013	−0.086419696
Jak2	31479.12631	31103.21718	34624.38581	31703.20444	27621.00875	32535.2277	−0.081628188
Stat5a	3039.268209	3541.281024	3588.384044	3100.228326	3508.798043	3015.940517	−0.079314866
Trim47	208.1690554	246.5979681	215.6830023	229.147311	215.5168688	191.8302302	−0.074981956
Nfatc1	628.4722912	671.8536477	587.819996	629.0318342	603.4472327	564.2065593	−0.071632279
Irf1	869.3536268	686.1127138	651.519121	763.8243701	614.4523494	729.3652067	−0.066447342
Birc2	1557.302791	1365.515279	1550.012043	1405.212187	1469.18308	1497.711958	−0.03285918
Nfkb1	3354.495636	2980.144832	3102.259142	3327.129094	3092.437794	2975.933143	−0.006343011
Zfp429	109.0409338	132.5254386	144.1611777	140.4088916	133.8955866	115.9188022	0.016717658
Cx3cl1	239.8900544	235.6939763	162.0416339	224.6542265	232.0245439	203.1143614	0.049304061
Gsap	1154.842617	872.3193428	989.0127309	1153.599453	920.7614311	1055.579181	0.053415042
Slc25a25	1165.74671	863.0928882	866.0845948	885.1376524	978.5382938	1181.756284	0.073121431
Ninj1	418.3206733	475.5817955	505.1228863	502.1021962	484.2251351	508.8117335	0.095857568
Hivep1	3843.197276	3880.982307	4014.162406	4860.39419	3838.951544	4017.150702	0.115472598
Phldb1	2838.038123	2498.691656	2065.192686	2919.381673	3060.339537	2693.829863	0.22872275
Mfhas1	1015.071966	830.3809128	830.3236825	1289.51526	1062.910855	826.819067	0.248726631
Ripk2	406.4252987	360.6704975	366.5493511	477.3902313	434.7021099	464.7010389	0.280342946
Mcc	4821.591837	4525.156591	4755.083808	5901.66653	6065.653491	5459.467834	0.305424051
Eno2	1815.035907	2503.724268	3294.474046	2865.464659	2987.889186	3592.457038	0.311165188
Rcl1	394.5299241	411.835382	528.590985	551.5261261	462.2149017	666.7895701	0.332124304
Sod2	3319.800794	3826.462348	3384.993855	4284.156099	4588.216573	4502.368343	0.344833311
Bid	804.9203477	795.1526317	630.2860793	854.8093318	1038.149343	943.7636992	0.346948979
Litaf	4232.770794	4561.22364	4487.994494	6373.440406	5310.885904	5248.146832	0.350321506
Tjp2	2611.034724	2471.851061	2520.026789	3533.810983	3335.467455	3001.578896	0.376623194
Stk10	254.7592726	320.4096048	297.2625835	392.0216252	386.0961778	355.9630474	0.378409795
Relb	485.727796	324.6034477	329.6709103	570.6217353	420.945714	490.3467916	0.37842541
Cdk6	1188.546178	1331.125766	984.5426168	1595.045008	1803.004954	1325.372499	0.430741462
Gadd45b	233.9423671	203.8207695	181.0396185	388.6518118	265.039894	230.8117743	0.515388199
Micall2	933.7869058	684.4351766	727.5110597	1125.517675	1399.484008	1092.509065	0.624958406
H2−q4	173.4742129	155.1721908	149.7488203	269.5850718	246.6980328	234.9150947	0.650990618
Stx11	435.172454	327.1197535	373.2545222	723.3866093	464.0490878	641.1438174	0.687336286
Bahcc1	79.30249731	76.32794249	69.28676759	94.35477513	179.7502395	92.32470971	0.704142499
Parp8	961.5427799	816.9606153	847.0866102	1274.912735	1491.193314	1653.638134	0.751321216
Cd44	4475.634692	4433.730813	5043.406163	8953.594197	8906.807787	8973.961784	0.943529946
Abtb2	280.5325842	217.2410671	238.0335725	486.3764004	511.7379268	434.9519657	0.961705604
Tnip1	1392.750109	1310.156551	1253.866988	2584.646876	2798.968016	2769.741291	1.043069505
Rasgef1b	256.741835	257.5019598	306.2028116	884.0143813	893.2486393	687.3061723	1.586854132

Supplementary Table 4: RelA target gene expression in OVE16 and OVE16-*Trp53*ko from spheroid RNA-seq data

RelA target genes	Normalized read counts						
Gene symbol	OVE16 (1)	OVE16 (2)	OVE16 (3)	OVE16-*Trp53*ko (1)	OVE16-*Trp53*ko (2)	OVE16-*Trp53*ko (3)	Log2(OVE16-*Trp53*ko/ OVE16)
Cxcl16	211.2848	163.2072	295.9268	0	0	4.642558	−7.174
Cxcl10	60.79422	129.5128	692.9531	2.94523	3.201752	3.481918	−6.51932
Slfn2	2.98988	14.7413	53.70913	0	0	1.160639	−5.94375
Ccl20	87.70314	82.13008	243.2708	3.926974	3.201752	11.60639	−4.46269
Il1rl1	91.68965	89.50073	75.82466	10.79918	13.87426	4.642558	−3.13209
Atp2b4	1107.252	1289.863	1231.098	178.6773	171.8274	206.5938	−2.70325
Rnd1	548.1446	520.1572	1267.957	118.791	85.38005	174.0959	−2.62672
Icam1	351.8092	267.4492	747.7154	72.64902	52.29528	103.2969	−2.58235
Gsdmd	16.94265	25.27079	56.86849	9.817435	3.201752	4.642558	−2.48799
Gch1	372.7384	394.8562	586.588	67.7403	66.16954	116.0639	−2.43757
Pdgfb	1709.215	1839.503	2382.158	435.8941	344.722	364.4408	−2.37283
Foxs1	9.966266	12.6354	5.265601	0.981743	2.134501	2.321279	−2.35755
Ccl7	185.3726	180.0544	1835.589	107.9918	108.8596	302.9269	−2.0822
Col27a1	69.76386	149.5189	108.4714	16.68964	17.07601	49.9075	−1.96978
Slc7a2	149.494	147.413	322.2548	35.34277	56.56429	71.95964	−1.91779
Cxcl5	1290.631	834.9892	13944.37	624.3889	581.6516	3791.809	−1.68499
Rgs16	290.0184	354.8441	1604.955	255.2533	149.4151	299.445	−1.67593
Cxcl2	439.5123	285.3494	2225.243	263.1073	262.5437	471.2196	−1.56529
Rab20	209.2916	259.0256	208.5178	83.4482	84.3128	76.6022	−1.46978
H2-k1	495.3234	471.7215	709.803	201.2574	136.6081	277.3928	−1.44649
Pdlim4	353.8025	414.8622	323.3079	142.3528	132.3391	136.9555	−1.40746
Lif	467.4179	471.7215	1236.363	198.3122	277.4852	446.8462	−1.2375
Fas	1467.034	1205.627	1810.314	643.042	593.3914	739.3273	−1.18205
Pim1	2932.076	2799.793	4356.758	1484.396	1326.593	1751.405	−1.14487
Ccl2	1384.314	1406.741	5735.293	808.9566	925.3063	2329.403	−1.06915
Cxcl1	7652.099	5283.702	30673.18	5369.155	4835.713	11099.19	−1.0335
Noct	556.1177	496.9923	595.0129	353.4277	197.4414	266.9471	−1.01098
Cdk6	4367.218	3920.132	3615.362	1787.755	2433.332	1695.694	−1.00841
Tnfaip3	4654.246	3995.944	7610.9	2389.564	2282.849	3746.544	−0.94971
Vcam1	18456.53	16153.3	28872.34	10529.2	9907.288	15119.65	−0.83625
Gsap	1478.994	1377.258	1912.466	823.6828	828.1865	1078.234	−0.80465
Angptl4	78.7335	44.22389	33.69985	24.54359	22.41226	45.26494	−0.76445
Bcl3	251.1499	228.4901	296.9799	165.9146	128.0701	165.9714	−0.75571
Jak2	12316.31	12197.37	15063.83	8079.749	7811.208	7988.681	−0.7289
Mt2	868.0618	908.6957	994.1455	740.2346	613.6691	394.6174	−0.66422
Ell2	1443.115	1406.741	1535.449	961.1269	959.4583	1000.471	−0.58619
Nfkb2	2707.835	2371.243	3282.576	1799.536	1802.586	2229.588	−0.51987
Psmb10	236.2005	194.7957	333.8391	165.9146	166.4911	203.1119	−0.51422

Clic4	38149.87	38262.09	38983.35	27924.71	29328.05	24932.86	−0.48963
Stk10	533.1952	610.7109	762.4591	492.8352	522.9528	350.5131	−0.48055
Cd44	22761.96	19707.01	30864.85	15324.03	17162.46	20441.18	−0.47046
Gadd45b	291.015	217.9606	386.4951	228.7462	157.9531	262.3045	−0.46442
Zc3h12a	597.976	380.1149	1319.56	457.4925	410.8915	832.1785	−0.43415
Rasgef1b	392.6709	309.5672	189.5616	279.7969	220.9209	162.4895	−0.42726
Traf3	1726.157	1822.656	2033.575	1360.696	1398.098	1431.068	−0.41398
Abtb2	794.3114	876.0542	984.6674	658.7499	737.4702	613.9783	−0.40139
Mfhas1	2331.11	2265.948	2565.401	1753.394	1849.545	1828.007	−0.39925
Tlr2	1086.323	847.6246	2306.333	877.6787	852.7333	1501.867	−0.39161
Nfkbib	293.0082	234.8078	370.6983	209.1114	203.8449	275.0715	−0.38507
Radil	4.983133	12.6354	11.58432	6.872204	8.538005	6.963837	−0.38428
Birc3	1027.522	850.7834	1490.165	788.34	768.4205	1052.7	−0.36834
Nfkbie	438.5157	461.192	641.3502	344.592	339.3857	518.8058	−0.35754
Csf1	4299.447	3266.25	7781.506	3280.005	3444.018	5515.359	−0.32645
Rcl1	737.5037	670.729	855.1336	639.115	570.9791	600.0506	−0.32237
Ripk2	863.0787	762.3356	1116.307	681.33	607.2656	904.1381	−0.32235
Rnf19b	1572.677	1617.331	2109.4	1446.108	1197.455	1614.449	−0.31565
Tnfaip2	6496.012	6060.779	9601.297	4944.06	4944.572	8095.46	−0.30111
Trim47	1103.266	998.1964	1160.539	1016.105	747.0755	892.5317	−0.29666
Nfkbiz	2788.561	2281.742	7760.443	2644.817	2499.501	5364.475	−0.28801
Cx3cl1	4160.916	3945.403	5970.139	3387.997	3846.371	4509.084	−0.26143
Serpina3g	1781.968	1745.791	2151.525	1395.057	1677.718	1693.373	−0.25289
Rel	312.9408	243.2314	347.5297	241.5089	263.6109	254.18	−0.25118
Stx11	599.9692	454.8743	741.3967	468.2916	447.178	598.8899	−0.24627
Uap1	2941.045	2886.135	2911.877	2548.606	2521.913	2347.974	−0.23635
Nfkb1	4313.4	4070.704	5632.087	3958.39	3699.091	4250.262	−0.23519
Micall2	876.0348	935.0194	1237.416	795.2122	916.7683	979.5797	−0.17964
Rhbdf2	306.961	259.0256	388.6014	290.5961	244.4004	321.4971	−0.15644
C9orf72	869.0584	841.3069	889.8866	863.9343	725.7304	769.5039	−0.14037
Mcc	5626.954	4874.105	6753.66	5432.968	5408.826	5102.171	−0.11398
Ninj1	393.6675	408.5445	431.7793	439.8211	350.0582	350.5131	−0.1138
Itpkc	467.4179	388.5385	362.2734	398.5879	323.377	422.4728	−0.09015
Nfkbia	6018.628	5537.463	8495.521	6445.146	5948.855	6904.644	−0.05522
Rsl1	160.4569	113.7186	128.4807	159.0424	117.3976	111.4214	−0.05401
Klhl25	260.1196	283.2435	225.3677	243.4724	254.0057	248.3768	−0.04358
Sod2	1819.84	1701.567	2945.577	2055.771	1962.674	2359.58	−0.01998
Tjp2	4886.46	4665.62	4847.512	4656.409	4717.248	4865.401	−0.01617
Zswim4	950.7818	937.1253	1397.491	963.0904	998.9466	1289.47	−0.01496
Zfp429	219.2579	195.8487	159.0212	183.586	181.4326	207.7545	−0.00341
Litaf	7012.265	7175.853	7883.658	7397.437	7546.529	7249.354	0.007923
Relb	612.9254	605.4461	813.0088	619.4801	583.7861	857.7125	0.020869
Hivep1	6046.534	4652.985	5681.584	5108.993	5929.645	5677.848	0.029239

Map3k8	375.7282	216.9077	611.8629	412.3323	300.9647	524.609	0.039469
Nfatc1	1765.026	2115.376	1675.514	1918.327	2038.449	1781.582	0.046613
Irf1	781.3553	764.4415	1107.883	892.4048	694.7802	1198.941	0.070266
Gbp2	1185.986	1015.044	2455.876	1467.707	1228.406	2334.046	0.111232
Phldb1	2783.578	3017.754	2987.702	3367.38	3191.079	3109.353	0.137485
Bahcc1	784.3452	675.9938	664.5189	707.8371	850.5988	810.1263	0.156645
Sqstm1	18222.32	16609.23	21214.05	21531.6	20575.53	21332.55	0.178784
Rab8b	1363.385	1326.717	1373.269	1524.648	1537.908	1630.698	0.207912
Tapbp	5396.733	4898.322	7343.407	6837.843	6569.995	7406.04	0.238821
Parp8	1662.373	1645.761	2428.495	2095.041	2009.633	2954.988	0.299396
Birc2	928.856	1017.149	1106.829	1225.216	1135.555	1418.301	0.307882
Camkk2	952.7751	1000.302	1359.578	1324.372	1214.531	1601.682	0.321847
Bid	1045.461	840.2539	1435.403	1307.682	1445.057	1500.707	0.356963
Tnip1	4320.376	4053.857	4740.094	5784.433	5810.113	6571.54	0.470104
Rrad	169.4265	192.6898	204.3053	273.9064	221.9881	327.3003	0.539358
Alpk1	793.3148	722.3235	1083.661	1293.938	1127.017	1530.883	0.604401
Stat5a	1764.029	1562.577	1266.904	2923.632	2600.89	2132.095	0.73711
Il15ra	19.93253	28.42964	27.38113	52.0324	41.62278	48.74686	0.91078
Eno2	304.9678	287.4553	393.867	888.4779	668.0989	1081.716	1.419521
Gem	1457.068	1314.081	1723.958	4208.734	4250.859	4940.842	1.575852

Supplementary Table 5: STAT1 target gene expression in OVE4 and OVE4-*Trp53*ko from spheroid RNA-seq data

**STAT1 target genes**	**Normalized read counts**						
**Gene symbol**	**OVE4 (1)**	**OVE4 (2)**	**OVE4 (3)**	**OVE4-*Trp53*ko (1)**	**OVE4-*Trp53*ko (2)**	**OVE4-*Trp53*ko (3)**	**Log2(OVE4-*Trp53*ko/OVE4)**
Icam1	2700.25	2517.983	2305.461	104.4642	77.03582	124.1254	−4.6216
Psmb8	249.8029	313.6995	283.8522	23.58869	21.09314	20.5166	−3.70006
Nckap5	95.163	80.52179	61.46407	4.493085	6.419651	13.33579	−3.28982
Tgm2	67799.67	68800.83	45915.89	5315.319	6102.337	8791.364	−3.17495
Ank3	15469.93	18941.91	14806.14	2390.321	2540.348	2697.933	−2.68969
Cyp1b1	968.4817	788.4425	677.2223	206.6819	151.3204	107.7122	−2.3859
C3	1733683	2095643	1646982	360035.4	340204.8	419987.2	−2.28941
F2rl1	177.4393	190.4005	176.5695	60.65664	27.51279	51.29151	−1.96483
Csf1	24600.63	27848.8	20020.52	6139.8	5741.003	7383.925	−1.91142
Cxcl10	25.77331	13.4203	6.705171	5.616356	1.834186	5.129151	−1.86736
Tgfb2	63154.53	71564.58	48302.93	22559.78	16710.35	18065.89	−1.6745
Tap1	123.9102	156.011	212.3304	52.79374	39.435	63.60147	−1.65942
Me3	507.536	611.4623	431.366	123.5598	243.9468	125.1513	−1.65395
Sdccag8	1100.322	1205.31	1385.735	407.7474	333.8219	460.5977	−1.61852
Wars	4829.522	6159.078	4559.516	1635.483	1997.429	1858.804	−1.50141
Dmd	549.1698	548.5547	462.6568	234.7637	231.1075	117.9705	−1.41825

Gbp2	1367.968	1607.081	1347.739	605.4431	447.5414	793.9925	−1.2268
Trim27	1038.863	1368.87	1518.721	669.4696	561.261	704.7453	−1.02054
Abcc4	10157.66	11772.12	12549.85	5518.631	6452.667	6380.663	−0.90981
Stat2	2148.106	1720.314	1233.751	821.1112	1039.066	904.7822	−0.88385
Alpk1	586.8385	596.3645	388.8999	240.38	295.304	322.1107	−0.87399
Tap2	664.1584	759.9244	695.1027	435.8292	387.0133	375.4538	−0.82253
Stat1	4427.062	4759.173	4819.9	2908.149	2871.418	2276.317	−0.79794
Casp7	457.9719	566.1688	506.2404	339.2279	255.869	294.4132	−0.78281
Arhgap6	498.6145	433.6434	480.5373	326.8719	284.2988	253.38	−0.70853
Ifi35	644.3328	712.9533	698.4553	432.4594	493.3961	481.1143	−0.54707
Bak1	2112.42	2320.873	1871.86	1246.831	1697.539	1391.026	−0.54037
Cald1	21170.79	22387.57	18483.92	13735.36	17599.02	11575.47	−0.53194
C4a	52.5379	22.64675	35.76091	19.09561	25.67861	32.82656	−0.51571
Irf7	433.1899	577.0728	496.1827	338.1046	399.8526	319.0332	−0.51119
Nrp2	745.4435	914.2578	1275.1	605.4431	718.0839	747.8301	−0.50269
Rdh10	5562.079	5947.708	6544.247	4866.011	3853.625	4234.627	−0.47889
Rnf19b	682.9928	774.1834	692.8677	585.2243	451.2098	507.7859	−0.47749
Nmi	227.0034	231.5001	227.9758	135.9158	191.6724	167.2103	−0.47238
Sh3kbp1	5257.756	6226.179	6157.582	4298.759	4656.999	4304.383	−0.41188
Pvr	1886.408	1571.014	1556.717	1445.65	1212.397	1358.199	−0.32015
Slc16a1	1381.846	1479.588	1449.434	1299.625	1059.242	1146.878	−0.29826
Scarb1	1137.991	1201.955	1211.401	994.095	1099.595	863.749	−0.26402
Gch1	1865.591	1787.416	1936.677	1996.053	1406.821	1313.063	−0.24522
Setbp1	3086.85	3179.772	2827.347	3275.459	2851.242	1766.479	−0.2043
Mark3	5123.933	4987.318	4834.428	4395.36	4553.367	4243.859	−0.18
Klf6	261.6982	233.1777	255.914	260.5989	186.1699	250.3025	−0.1071
Zc3hav1	3292.045	3625.997	3485.571	3338.362	3234.587	3266.243	−0.08047
Znf280d	1628.675	1188.535	1167.817	1317.597	1285.764	1305.882	−0.0277
Irf9	403.4515	453.7738	402.3103	433.5827	319.1484	491.3726	−0.01779
Psd3	3380.269	3176.417	3309.002	3032.832	3721.564	3118.524	0.001057
Orai1	343.9746	272.5998	251.4439	275.2014	331.9877	273.8966	0.021557
Pml	772.2081	895.8049	987.8952	899.7402	818.9641	984.7969	0.025624
Acer3	1748.62	1776.512	1766.813	2020.765	1773.658	1699.8	0.054118
Lyn	2084.664	2278.934	2476.443	2698.097	2662.321	2014.73	0.108667
Nek1	1422.489	1616.307	2023.844	2161.174	1673.695	1931.638	0.187808
Lins	338.0269	330.4748	356.4916	408.8707	418.1944	412.3837	0.274084
Sod2	3319.801	3826.462	3384.994	4284.156	4588.217	4502.368	0.344833
Plscr1	870.3449	785.9262	1006.893	1122.148	1180.299	1251.513	0.416286
Bcl2l11	1103.296	987.2306	1153.289	1504.06	1563.644	1683.387	0.550567
Bcl6	1304.526	1120.595	1144.349	1928.657	1898.383	1935.741	0.691055
Il4r	2477.212	1845.291	1599.183	3267.596	3436.348	3713.505	0.814922
Fyn	1140.965	1047.622	1147.702	2679.002	2362.432	2069.099	1.091714
C1s	243.8552	126.6541	65.93418	370.6795	232.9416	442.1328	1.260677

Tcaf2	39.65125	25.16306	18.99798	85.36861	72.45035	60.52398	1.381362
H2-t23	57.49431	36.06705	65.93418	179.7234	212.7656	152.8487	1.773634
Dtna	29.73844	48.64858	49.17125	174.107	228.3562	292.3616	2.445493
Plscr4	167.5265	80.52179	97.22498	1076.094	981.2896	768.3468	3.032813

Supplementary Table 6: STAT1 target gene expression in OVE16 and OVE16-*Trp53*ko from spheroid RNA-seq data

**STAT1 target genes**	**Normalized read counts**						
**Gene symbol**	**OVE16 (1)**	**OVE16 (2)**	**OVE16 (3)**	**OVE16-*Trp53*ko (1)**	**OVE16-*Trp53*ko (2)**	**OVE16-*Trp53*ko (3)**	**Log2(OVE16-*Trp53*ko/ OVE16)**
Cxcl10	60.79422	129.5128	692.9531	2.94523	3.201752	3.481918	−6.51932
F2rl1	1969.334	2443.896	1430.137	73.63076	56.56429	71.95964	−4.85327
Arhgap6	53.81784	76.86533	61.08097	10.79918	6.403504	2.321279	−3.29602
Icam1	351.8092	267.4492	747.7154	72.64902	52.29528	103.2969	−2.58235
Gch1	372.7384	394.8562	586.588	67.7403	66.16954	116.0639	−2.43757
Tcaf2	124.5783	85.28893	72.6653	33.37928	20.27776	10.44575	−2.13996
Ank3	39.86507	60.01814	46.33729	12.76267	14.94151	16.24895	−1.73411
Irf7	274.8199	629.7659	172.4164	78.97655	85.88732	172.4164	−1.675
Tap1	75.74362	122.1422	165.3399	40.25148	37.35377	51.06814	−1.49715
Me3	115.6087	76.86533	132.6932	46.14194	42.69003	51.06814	−1.21678
Ccl2	1384.314	1406.741	5735.293	808.9566	925.3063	2329.403	−1.06915
Sh3kbp1	4505.749	4846.728	4636.888	2367.965	2615.831	2273.693	−0.94679
Pvr	1508.893	1392	1776.614	865.8978	715.0579	872.8009	−0.93075
Acer3	1379.331	1243.534	1221.619	762.8147	803.6397	702.1869	−0.76096
Dmd	1556.731	1335.14	1007.836	796.194	870.8765	672.0102	−0.73742
Casp7	317.9239	359.0559	255.9082	217.9471	208.1139	154.365	−0.68459
Scarb1	746.4733	762.3356	689.7938	453.5655	453.5815	474.7015	−0.66999
Gbp5	8.96964	9.476548	64.24034	13.74441	11.73976	30.17663	−0.57099
Lyn	2182.612	2215.406	2309.493	1536.429	1690.525	1388.125	−0.53942
Stat1	1948.405	2203.824	3071.952	1646.384	1857.016	1552.936	−0.51474
Klf6	422.5697	365.3736	472.851	281.7604	288.1577	341.228	−0.46858
Pml	1404.247	1522.565	1989.344	1091.699	1242.28	1240.724	−0.45971
Abcc4	6412.296	5232.107	7729.903	3906.357	4326.634	5908.815	−0.45418
Bak1	1188.976	1181.41	1160.539	901.2405	969.0636	727.7209	−0.44263
Stat2	492.3336	518.0513	1009.942	551.7398	453.5815	506.0388	−0.41874
Irf9	550.1379	618.0815	828.8056	524.251	490.9353	485.1473	−0.41257
Psmb8	60.01814	67.3997	61.79085	42.69003	39.46174	61.79085	−0.39448
H2-t23	1101.272	1197.204	1510.174	1020.031	897.5578	1068.949	−0.35081
Csf1	4299.447	3266.25	7781.506	3280.005	3444.018	5515.359	−0.32645
Rnf19b	1572.677	1617.331	2109.4	1446.108	1197.455	1614.449	−0.31565
Trim27	1052.438	1071.903	926.7458	788.34	837.7918	842.6242	−0.30554
Wars	1595.599	1614.172	1547.034	1350.879	1294.575	1204.744	−0.30506

Il4r	4492.793	4507.678	5399.348	3818	3689.486	4153.929	−0.30431
Zc3hav1	2749.693	2430.208	3127.767	2180.452	2328.741	2270.211	−0.29329
Mark3	3350.659	3474.734	3220.442	2990.391	2690.539	2671.792	−0.26628
Nrp2	4973.167	4416.071	6979.028	4161.611	4826.107	5025.569	−0.22411
Ifi35	358.0029	478.1166	315.9306	297.7629	378.3685	315.9306	−0.2157
Fyn	1620.515	1631.019	1693.417	1527.593	1525.101	1573.827	−0.09603
Plscr1	1027.522	1058.215	1423.819	993.5244	1062.982	1284.828	−0.07086
Tgm2	3647.653	4298.141	3659.593	4270.584	4758.871	2173.878	−0.05087
Orai1	218.2612	181.1074	188.5085	210.0931	187.8361	177.5778	−0.03068
Sod2	1819.84	1701.567	2945.577	2055.771	1962.674	2359.58	−0.01998
Lins	347.8227	312.7261	385.442	317.1031	322.3097	406.2238	−0.00049
Bcl2l11	1477.001	1392	1591.265	1433.345	1337.265	1692.212	0.000827
Nmi	146.5041	148.4659	171.6586	161.0059	149.4151	159.0076	0.008631
Slc16a1	765.4093	773.9181	722.4405	769.6869	741.7392	854.2306	0.06479
Nek1	685.6791	746.5414	571.8443	751.0338	685.1749	705.6688	0.095947
Gbp2	1185.986	1015.044	2455.876	1467.707	1228.406	2334.046	0.111232
Il7	26.90892	45.27684	26.32801	38.288	34.15202	38.3011	0.168794
Cyp1b1	58036.56	42727.65	54610.6	57695.1	63976.34	57558.43	0.206058
Znf280d	1315.547	1306.711	1306.922	1601.224	1451.461	1558.739	0.230984
Psd3	6866.758	5237.372	5157.13	6294.939	8086.558	6877.949	0.300566
Tap2	283.042	237.9666	274.8644	358.3364	335.1167	315.6939	0.342526
Setbp1	2989.88	2677.651	2247.359	3195.575	4131.327	2994.45	0.382991
Sdccag8	244.1735	276.9258	208.5178	313.1762	304.1664	340.0674	0.391997
Rdh10	6959.444	6281.898	8382.837	9666.246	10677.84	11267.49	0.547808
Plscr4	1989.267	1825.815	1835.589	2637.945	2939.208	2736.788	0.55711
Bcl6	2015.179	1980.599	2188.384	2969.774	2937.074	3262.557	0.56825
Alpk1	793.3148	722.3235	1083.661	1293.938	1127.017	1530.883	0.604401
Cald1	14130.17	13583.05	14294	22660.6	23229.78	20184.68	0.653468
Tgfb2	22850.66	18625.63	12557.41	29554.41	32261.92	26045.91	0.701384
C1s	183.3793	125.301	784.5746	332.811	334.0495	1334.735	0.872521
Mctp1	43.85157	57.91224	114.7901	132.5354	173.9619	136.9555	1.034054
C3	57814.31	33624.9	101083.7	124974	133946.4	189779.6	1.220721
Dtna	247.1634	168.472	260.1207	493.817	488.8008	784.5923	1.386899

Supplementary Table 7: STAT3 target gene expression in OVE4 and OVE4-*Trp53*ko from spheroid RNA-seq data

**STAT3 target genes**	**Normalized read counts**						
**Gene symbol**	**OVE4 (1)**	**OVE4 (2)**	**OVE4 (3)**	**OVE4-*Trp53*ko (1)**	**OVE4-*Trp53*ko (2)**	**OVE4-*Trp53*ko (3)**	**Log2(OVE4-*Trp53*ko/OVE4)**
Tnfrsf1b	378.6694	452.0963	546.4714	22.46542	13.7564	7.180811	−4.98785
Icam1	2700.25	2517.983	2305.461	104.4642	77.03582	124.1254	−4.6216
Lcn2	60862.68	71044.54	47374.27	1988.19	1724.135	4015.099	−4.5361

Muc1	171.4917	212.2085	197.8025	19.09561	11.00512	31.80073	−3.23174
Cdkn1a	2282.921	3377.721	3999.635	440.3223	460.3807	532.4058	−2.75292
Nos2	1827.923	1720.314	1296.333	399.8845	178.8331	793.9925	−1.81934
Rora	143.7358	197.1106	175.452	37.06795	40.35209	82.06641	−1.69477
Bcl2	926.8479	746.5041	617.9933	263.9687	284.2988	197.9852	−1.61846
Ptgs2	739.4958	877.352	1353.327	439.199	238.4442	403.1512	−1.45846
Hsp90b	70322.48	73237.92	75304.66	37529.61	32562.31	32281.85	−1.0962
Ccnd1	1088.427	1020.781	1339.917	589.7173	867.57	263.6383	−1.00305
Stat1	4427.062	4759.173	4819.9	2908.149	2871.418	2276.317	−0.79794
Cdc25a	980.3771	975.4879	883.9651	507.7186	615.3694	638.0663	−0.68928
Irf4	503.5709	594.6869	700.6904	414.487	473.22	430.8486	−0.4482
Timp1	1328.317	1338.675	961.0745	720.0168	995.046	1068.915	−0.38205
Vegfa	4948.476	4782.659	5459.127	4374.018	3661.953	3650.929	−0.37825
Hsp90a	14874.17	15007.25	13801.48	12096.51	12414.69	13904.1	−0.18539
Pim1	1100.322	891.611	886.2001	894.1238	857.482	813.4833	−0.16612
Akt1	5442.134	5833.636	5516.121	5064.83	5240.27	5253.276	−0.11007
Rhou	4886.025	4656.004	4443.293	5147.952	4442.399	4092.036	−0.03159
Foxo3a	1331.291	1808.385	2369.16	1791.617	1869.036	1896.76	0.012666
Il6ra	1091.401	1043.428	1266.16	1169.325	1360.049	1039.166	0.06938
Hif1a	21323.45	20314.98	17649.13	22030.72	25762.98	23752.07	0.271137
Pik3r1	2017.257	2124.601	1802.573	2225.2	3210.743	1930.612	0.309451
Vimentin	13831.35	12495.97	13363.41	27761.65	15321.87	12695.67	0.490925
Rorc	321.1751	226.4675	262.6192	483.0066	263.2057	424.6937	0.531165
Tgfb1	1131.052	1018.265	958.8395	1548.991	1535.214	1439.24	0.541363
Fos	2181.81	1559.271	1726.582	4278.54	2187.267	1786.996	0.59396
Foxo1	1696.082	1640.631	1982.496	2998.011	2606.378	2647.668	0.633542
Zeb1	1233.154	1148.274	1250.514	2028.628	2112.065	1862.907	0.725087
Hspa1a	20.81691	14.25907	11.17529	44.93085	22.01023	12.30996	0.776937
Birc5	45.59894	27.67936	22.35057	26.95851	125.6417	81.04058	1.288774
Twist	23.79075	19.29168	31.2908	98.84786	82.53838	83.09224	1.830295
Myc	210.1516	136.7193	151.9839	340.3512	692.4053	2226.051	2.707652

Supplementary Table 8: STAT3 target gene expression in OVE16 and OVE16-*Trp53*ko from spheroid RNA-seq data

STAT3 target genes	Normalized read counts						
**Gene symbol**	**OVE16 (1)**	**OVE16 (2)**	**OVE16 (3)**	**OVE16-*Trp53*ko (1)**	**OVE16-*Trp53*ko (2)**	**OVE16-*Trp53*ko (3)**	**Log2(OVE16-*Trp53*ko/ OVE16)**
Birc5	259.1229	354.8441	249.5895	6.872204	3.201752	11.60639	−5.31583
Lcn2	609.9355	496.9923	4446.274	98.17435	108.8596	372.5653	−3.26019
Nos2	494.3268	355.897	2986.649	60.8681	71.50579	345.8706	−3.00411
Ccnd1	6643.513	9660.814	6150.222	1161.403	1525.101	753.255	−2.70663
Icam1	351.8092	267.4492	747.7154	72.64902	52.29528	103.2969	−2.58235

Myc	809.2608	935.0194	771.9371	333.7928	360.7307	312.212	−1.32157
Pim1	2932.076	2799.793	4356.758	1484.396	1326.593	1751.405	−1.14487
Vimentin	40841.76	47681.78	28791.25	23890.73	23605.45	18887.09	−0.82149
Ptgs2	604.9524	463.2979	3937.617	612.6079	483.4645	1894.164	−0.74336
Timp1	1477.997	1559.419	1503.856	983.707	1048.04	911.102	−0.62588
Cdc25a	705.6117	767.6004	797.212	512.4701	499.4733	489.7898	−0.59633
Fscn1	1305.581	1582.584	1414.34	967.0173	974.3999	1048.057	−0.52528
Stat1	1948.405	2203.824	3071.952	1646.384	1857.016	1552.936	−0.51474
Hsp90a	9600.504	10129.38	10491.18	7568.261	7481.427	8806.932	−0.34116
Bcl2	1489.957	1229.845	1778.72	1223.252	1059.78	1291.792	−0.33158
Twist	369.7485	446.4507	481.276	360.2999	357.529	349.3525	−0.2819
Rorc	32.88868	28.42964	25.27489	23.56184	22.41226	29.01599	−0.20755
Akt1	7264.412	7672.845	7145.421	6715.125	6666.048	6073.626	−0.18279
Muc1	31.89205	25.27079	29.48737	18.65313	26.68127	31.33726	−0.17651
Hsp90b	23741.64	25050.73	22447.26	21651.37	21925.6	20183.52	−0.16002
Tgfb1	3226.08	3443.146	2820.256	2907.924	3163.331	2617.242	−0.12722
Hspa1a	53.81784	62.12404	49.49665	61.84984	42.69003	48.74686	−0.11006
Hif1a	16079.57	13101.85	20951.83	16644.48	16814.53	17370.13	0.019888
Foxo3a	1131.171	999.2493	945.702	1127.042	1054.444	948.2424	0.024924
Zeb1	3122.431	2994.589	2885.549	3094.455	3296.737	2775.089	0.026
Foxo1	3233.057	2929.306	3096.174	3280.005	3589.164	3346.123	0.141874
Il6ra	1022.539	909.7486	1315.347	1159.439	1220.935	1352.145	0.20076
Pik3r1	6352.498	6743.09	6769.457	7543.717	9457.975	7101.953	0.279019
Fos	1763.033	1412.006	1524.918	2278.627	1884.765	1571.506	0.28712
Cdkn1a	2644.05	2234.359	2145.206	2803.859	2661.723	3552.717	0.360642
Vegfa	3765.255	4245.493	3183.583	6241.925	4273.272	3940.371	0.368857
Irf4	54.81446	41.06504	67.3997	71.66727	68.30404	71.95964	0.376253
Rhou	1839.773	1920.58	1702.895	2300.225	2480.291	2791.338	0.470887
Fgf2	65.77736	61.07109	61.08097	100.1378	143.0116	73.12028	0.750964
Tnfrsf1b	1181.999	1201.416	1272.169	2238.375	2447.206	2249.319	0.923773

Supplementary Table 9: STAT5 target gene expression in OVE4 and OVE4-*Trp53*ko from spheroid RNA-seq data

STAT5 target genes	Normalized read counts						
Gene symbol	OVE4 (1)	OVE4 (2)	OVE4 (3)	OVE4-*Trp53*ko (1)	OVE4-*Trp53*ko (2)	OVE4-*Trp53*ko (3)	Log2(OVE4-*Trp53*ko/OVE4)
Nckap5	95.163	80.52179	61.46407	4.493085	6.419651	13.33579	−3.28982
Adamts9	6962.759	7854.229	7697.536	885.1377	1197.724	1064.812	−2.8385
Cyp1b1	968.4817	788.4425	677.2223	206.6819	151.3204	107.7122	−2.3859
Ctgf	6415.572	3768.587	3064.263	1616.387	918.0102	472.9077	−2.13928
Mapkapk3	791.0424	1154.984	1121.999	263.9687	298.0552	399.0479	−1.67459
Cish	841.5978	852.1889	837.0289	232.5171	297.1382	373.4022	−1.48671

Socs3	1055.714	951.1636	923.0785	287.5574	221.9365	623.7047	−1.37048
Mvd	257.7331	240.7266	151.9839	64.02645	149.4862	94.37637	−1.07901
Mpp1	2392.953	2427.396	2114.364	1122.148	1128.024	1149.956	−1.02825
Trim7	629.4636	629.0764	677.2223	265.092	263.2057	455.4686	−0.97651
Sbno2	5679.05	5166.815	4921.596	2552.072	2129.49	3397.549	−0.96468
Cln6	905.0398	1056.848	1005.776	520.0745	605.2814	596.0073	−0.78578
Fn1	168753.7	146244.3	144903.2	126420.8	95159.41	81931	−0.59957
Ergic2	4229.797	4197.198	4080.097	3132.803	2821.895	2889.763	−0.4999
Slc25a37	328.1141	257.502	254.7965	234.7637	178.8331	220.5535	−0.40627
Zfp36	368.7566	345.5727	273.7945	370.6795	160.4913	250.3025	−0.3385
Gclm	1849.731	1732.057	1795.868	1579.319	1496.696	1392.051	−0.26733
Flot1	2162.976	2734.386	2610.547	2009.532	1858.948	2399.417	−0.26043
Tlcd2	48.57278	68.77903	64.81665	44.93085	57.77686	50.26568	−0.25199
Snrpf	458.9632	478.9369	423.5433	362.8166	403.5209	412.3837	−0.20791
Atg16l2	367.7653	260.857	270.4419	235.8869	232.9416	316.9815	−0.19424
Hsp90aa1	14874.17	15007.25	13801.48	12096.51	12414.69	13904.1	−0.18539
Ppm1k	438.1463	424.4169	505.1229	472.8971	289.8014	441.1069	−0.18413
Pofut2	2134.228	2474.367	2257.408	2113.996	2088.221	2045.505	−0.13614
Fbln2	3063.059	2937.368	2640.72	2720.563	2723.766	2551.239	−0.11202
Zfp810	613.6031	601.3971	616.8757	498.7324	652.0532	558.0516	−0.10031
Plec	28850.25	30665.38	25781.38	30694.51	26478.31	22642.12	−0.09584
Agpat3	3299.975	3498.504	3348.115	2999.134	3563.824	3058	−0.07674
Trim41	982.3597	989.7469	955.4869	901.9867	970.2845	944.7895	−0.05552
Birc2	1557.303	1365.515	1550.012	1405.212	1469.183	1497.712	−0.03286
Psmd3	1086.444	1373.064	1382.383	1096.313	1366.469	1297.675	−0.03091
Wdr81	1807.106	2056.661	1736.639	1638.853	2021.273	1828.029	−0.02921
Plekhg2	467.8847	494.0347	495.0651	476.267	457.6294	495.4759	−0.0276
Irf2	792.0337	910.0639	942.0765	969.383	758.436	883.2397	−0.01818
Necap1	1227.206	1264.024	1282.923	1288.392	1227.071	1216.635	−0.01617
Clcn7	3629.081	3465.792	2895.516	2978.915	3730.735	3210.848	−0.01013
Sox6	603.6903	687.7903	555.4117	477.3902	738.2599	651.4021	0.015663
Poli	303.3321	275.1161	334.141	296.5436	294.3869	335.4464	0.021634
Afap1l2	1374.907	1644.825	1354.445	1358.035	1556.307	1564.391	0.034079
Hcfc2	1008.133	906.7089	1039.302	1042.396	1044.569	954.022	0.0418
Serpinh1	8722.283	9626.547	8851.943	8127.99	10574.08	9733.076	0.064028
Katnb1	262.6895	280.1487	308.4379	262.8454	304.4749	331.3431	0.078154
Nacc2	1608.849	1794.965	1705.349	1799.48	2312.909	1764.428	0.201948
Sgk1	32246.38	32384.02	41365.32	41218.43	35452.98	48584.34	0.240871
Tbc1d19	1120.148	970.4553	987.8952	1281.652	1161.04	1223.815	0.25218
Cavin1	5890.193	6386.384	6520.779	8291.988	8059.414	6253.46	0.266103
Stk11	1986.528	2226.092	2259.643	2816.041	2492.659	2540.981	0.278364
Pds5a	5458.986	5375.668	5263.559	7055.266	6862.607	6837.158	0.366561
Ubc	3745.06	3793.75	4137.091	4960.365	5320.974	4903.468	0.379095

Rhoq	9044.45	9748.169	8616.145	12777.21	12463.29	11584.7	0.426056
Arhgap24	2102.507	2639.605	2638.485	3190.09	3479.451	3292.915	0.432764
Ugp2	5420.326	5610.523	5966.485	8262.782	7970.456	7295.704	0.469128
Cuedc1	571.9693	528.4242	571.0571	782.92	807.959	776.5534	0.502223
Ptgr2	1596.954	1890.584	1943.382	2418.403	2854.911	2444.553	0.507005
Trib3	152.6573	211.3697	210.0954	361.6933	292.5527	235.9409	0.63275
Serpine1	648.2979	1042.589	1342.152	1919.67	1381.142	1556.184	0.6793
Bcl6	1304.526	1120.595	1144.349	1928.657	1898.383	1935.741	0.691055
Tle1	999.2115	1092.915	1306.391	1992.683	1785.58	1756.221	0.703543
Lgals3	10949.69	11380.41	11921.79	21817.3	18262.99	17127.26	0.740021
St3gal5	320.1838	369.0582	394.4876	780.6734	1190.387	634.9888	1.265859
Msln	376.6869	350.6053	457.0692	1043.519	1157.371	1118.155	1.486659
Socs2	166.5352	107.3624	127.3983	483.0066	496.1473	551.8966	1.931784

Supplementary Table 10: STAT5 target gene expression in OVE16 and OVE16-*Trp53*ko from spheroid RNA-seq data

**STAT5 target genes**	**Normalized read counts**						
**Gene symbol**	**OVE16 (1)**	**OVE16 (2)**	**OVE16 (3)**	**OVE16-*Trp53*ko (1)**	**OVE16-*Trp53*ko (2)**	**OVE16-*Trp53*ko (3)**	**Log2(OVE16-*Trp53*ko/ OVE16)**
Serpine1	15034.11	17825.39	12262.53	3326.147	2441.87	1854.702	−2.56545
Msln	193.3456	243.2314	202.1991	59.88635	67.23679	111.4214	−1.42105
Socs2	802.2844	867.6306	766.6715	381.8982	358.5962	237.9311	−1.31633
Trib3	91.68965	98.97728	119.0026	60.8681	49.09353	39.46174	−1.05132
Cish	255.1364	279.0317	399.1326	153.152	160.0876	159.0076	−0.9828
Gclm	680.696	658.0936	838.2837	377.9712	341.5202	421.3121	−0.93234
Mvd	102.6525	121.0892	96.88706	61.84984	60.83329	49.9075	−0.89355
Ctgf	104404.6	91913.04	92173.3	62491.9	55087.21	51344.37	−0.77216
Mpp1	1536.798	1444.647	1281.647	916.9484	985.0724	927.3509	−0.59142
Lgals3	9365.3	9432.324	9384.355	7075.425	6321.326	6357.983	−0.51257
Adamts9	1059.414	1177.198	1014.155	728.4537	1002.148	695.223	−0.4223
Socs3	628.8714	646.5112	1041.536	530.1415	473.8593	727.7209	−0.42
Hsp90aa1	9600.504	10129.38	10491.18	7568.261	7481.427	8806.932	−0.34116
Snrpf	274.0723	274.8199	254.8551	219.9105	276.4179	156.6863	−0.29963
Psmd3	1348.436	1290.916	1282.7	1084.827	1148.362	1059.664	−0.25227
Trim7	72.75374	82.13008	69.50594	55.95938	61.90054	73.12028	−0.23258
Slc25a37	789.3283	840.2539	1610.221	823.6828	752.4117	1265.097	−0.18941
Ergic2	2892.21	3054.607	2915.037	2695.868	2725.758	2531.355	−0.15611
Serpinh1	8296.917	9412.318	8749.323	8480.3	8663.941	7031.154	−0.13019
Sbno2	2905.167	2479.697	3832.305	2889.271	2381.036	3216.132	−0.11916
Ubc	3903.787	4396.065	4849.619	4266.657	3941.357	3936.889	−0.11465
Ugp2	3487.197	3583.188	3074.058	3193.612	3213.492	2975.88	−0.11257

Plec	30472.86	22041.4	21175.09	21560.07	29334.45	18454.17	−0.08759
Zfp810	590.9996	641.2464	659.2533	564.5025	613.6691	602.3719	−0.08721
Pds5a	5726.617	5512.192	5422.516	5308.287	5257.277	5475.897	-0.0547
Necap1	925.8661	998.1964	888.8335	933.6381	903.9613	889.0498	-0.04493
Arhgap24	1287.642	935.0194	777.2027	914.9849	974.3999	1022.523	-0.04293
Fbln2	14581.64	14329.59	9412.789	14074.27	15459.13	8970.582	0.006759
Katnb1	402.6372	453.8214	474.9572	484.9813	423.6985	446.8462	0.025892
Cln6	407.6203	445.3978	366.4858	442.7663	377.8067	457.2919	0.067441
Mapkapk3	190.3557	177.9485	155.8618	170.8234	177.1636	201.9513	0.069246
Trim41	713.5847	869.7365	780.3621	816.8106	857.0023	828.6966	0.082339
Fn1	954912.8	654608.3	506020.1	728996.6	918409.7	653130.1	0.120943
Pofut2	2038.101	2216.459	2113.612	2229.539	2395.978	2314.315	0.124021
Poli	130.5581	165.3131	170.6055	185.5495	165.4239	167.1321	0.151441
Irf2	947.7919	820.2479	944.6489	1044.575	964.7946	1013.238	0.15607
Agpat3	1909.537	1847.927	1761.87	2137.256	2159.048	1898.806	0.166636
Tbc1d19	1119.212	1204.575	1105.776	1329.281	1215.599	1326.611	0.174864
Rhoq	8735.432	9111.174	9322.22	10566.51	10402.49	10067.39	0.192008
Cyp1b1	58036.56	42727.65	54610.6	57695.1	63976.34	57558.43	0.206058
Plekhg2	994.6334	1019.255	879.3554	1145.695	1133.42	1059.664	0.206632
Ppm1k	501.3032	418.0211	486.5416	533.0867	576.3154	517.6452	0.210797
Cavin1	18245.24	18136.01	15260.77	21805.5	21259.63	18244.09	0.247559
Stk11	1886.614	1858.456	2018.832	2357.166	2166.519	2450.11	0.274898
Hcfc2	576.0502	575.9635	545.5163	714.7093	648.8884	706.8294	0.286491
Birc2	928.856	1017.149	1106.829	1225.216	1135.555	1418.301	0.307882
Ptgr2	1206.915	1328.823	1181.601	1646.384	1670.247	1429.908	0.352606
Tle1	1510.886	1583.636	1678.674	2008.647	2000.028	2140.219	0.365371
Gm4262	32.88868	53.70044	38.96545	51.05066	49.09353	64.99581	0.395375
Clcn7	1785.955	1697.355	2119.931	2331.641	2431.197	2780.892	0.429016
Flot1	1397.271	1349.882	1617.593	1946.797	1901.841	2034.601	0.430713
Afap1l2	665.7466	658.0936	460.2135	804.0479	913.5666	692.9017	0.434183
Zfp36	357.789	270.6081	360.1671	448.6568	397.0172	511.842	0.457563
Wdr81	1285.648	1057.162	1343.781	1707.252	1813.259	1775.778	0.522694
Bcl6	2015.179	1980.599	2188.384	2969.774	2937.074	3262.557	0.56825
Sgk1	12123.96	12812.29	16790.95	18989.86	22769.79	23687.49	0.649342
Nacc2	3332.719	3198.861	3174.104	4963.695	5509.148	4831.742	0.657062
Atg16l2	130.5581	154.7836	213.7834	224.8193	210.2484	362.1195	0.675517
Cuedc1	949.7852	963.449	844.6024	1651.293	1585.934	1381.161	0.743852
Sox6	361.7755	312.7261	243.2708	511.4884	665.9644	408.5451	0.789183
St3gal5	826.2035	962.3961	688.7406	2631.073	3308.477	2155.307	1.708214
